# Genetic underpinnings of sociability in the general population

**DOI:** 10.1038/s41386-021-01044-z

**Published:** 2021-05-30

**Authors:** Janita Bralten, Nina R. Mota, Cornelius J. H. M. Klemann, Ward De Witte, Emma Laing, David A. Collier, Hilde de Kluiver, Stephanie E. E. C. Bauduin, Celso Arango, Jose L. Ayuso-Mateos, Chiara Fabbri, Martien J. Kas, Nic van der Wee, Brenda W. J. H. Penninx, Alessandro Serretti, Barbara Franke, Geert Poelmans

**Affiliations:** 1 Department of Human Genetics, Radboud University Medical Center, Nijmegen, The Netherlands; 2 Donders Institute for Brain, Cognition and Behaviour, Nijmegen, The Netherlands; 3 Lilly Research Centre, Eli Lilly and Company, Surrey, UK; 4 Department of Psychiatry, Amsterdam University Medical Center/GGZ in Geest, Vrije Universiteit, Amsterdam, The Netherlands; 5 Department of Psychiatry, Leiden Institute for Brain and Cognition/Psychiatric Neuroimaging, Leiden University Medical Center, Leiden, The Netherlands; 6 Institute of Psychiatry and Mental Health, Department of Child and Adolescent Psychiatry, Hospital General Universitario Gregorio Marañón School of Medicine, Universidad Complutense, CIBERSAM, Instituto de Investigación Sanitaria Gregorio Marañón (IiSGM), Madrid, Spain; 7 Department of Psychiatry, Instituto de Investigación Sanitaria La Princesa (IIS-IP), CIBERSAM, Universidad Autónoma de Madrid, Madrid, Spain; 8 Institute of Psychiatry, Psychology and Neuroscience, King’s College London, London, UK; 9 Department of Biomedical and NeuroMotor Sciences, University of Bologna, Bologna, Italy; 10 Groningen Institute for Evolutionary Life Sciences, University of Groningen, Groningen, The Netherlands; 11 Department of Psychiatry, Radboud University Medical Center, Nijmegen, The Netherlands

**Keywords:** Bipolar disorder, Schizophrenia, Depression, Behavioural genetics, Autism spectrum disorders

## Abstract

Levels of sociability are continuously distributed in the general population, and decreased sociability represents an early manifestation of several brain disorders. Here, we investigated the genetic underpinnings of sociability in the population. We performed a genome-wide association study (GWAS) of a sociability score based on four social functioning-related self-report questions from 342,461 adults in the UK Biobank. Subsequently we performed gene-wide and functional follow-up analyses. Robustness analyses were performed in the form of GWAS split-half validation analyses, as well as analyses excluding neuropsychiatric cases. Using genetic correlation analyses as well as polygenic risk score analyses we investigated genetic links of our sociability score to brain disorders and social behavior outcomes. Individuals with autism spectrum disorders, bipolar disorder, depression, and schizophrenia had a lower sociability score. The score was significantly heritable (SNP *h*^2^ of 6%). We identified 18 independent loci and 56 gene-wide significant genes, including genes like ARNTL, DRD2, and ELAVL2. Many associated variants are thought to have deleterious effects on gene products and our results were robust. The sociability score showed negative genetic correlations with autism spectrum, disorders, depression, schizophrenia, and two sociability-related traits—loneliness and social anxiety—but not with bipolar disorder or Alzheimer’s disease. Polygenic risk scores of our sociability GWAS were associated with social behavior outcomes within individuals with bipolar disorder and with major depressive disorder. Variation in population sociability scores has a genetic component, which is relevant to several psychiatric disorders. Our findings provide clues towards biological pathways underlying sociability.

## Introduction

Sociability, the inclination to seek or enjoy social interaction, is a human trait that shows significant variability and is continuously distributed in the general population [1]. Difficulties with sociability include a tendency to avoid social contacts and activities, and to prefer being alone rather than being with others, potentially linked to a desire to avoid social embarrassment [2]. Reduced sociability has been associated with adverse physical and mental health outcomes [3, 4], including psychiatric and neurological disorders [5–7]. Deficits in social interaction are key features of autism spectrum disorders (ASDs) [8]. In addition, reduced sociability is a common feature of bipolar disorder (BPD) [9], major depressive disorder (MDD) [6, 10, 11], schizophrenia (SCZ) [12, 13], and Alzheimer’s disease (AD) [7, 14, 15]. Reduced sociability thus represents a behavior that is shared by these disorders, of which ASD and AD can co-occur with MDD, SCZ, and BPD (e.g., [16–18]). More specifically, in subgroups of patients, social withdrawal—the gradual withdrawal from friends, family, and colleagues—is one of the earliest signs of these disorders, making it a potential target phenotype for early intervention and prevention [19, 20].

Sociability is a complex behavioral trait modulated by multiple factors, such as temperament and personality, disability status, aging, and socioeconomic status [4]. Beyond those influences, there is also growing evidence that sociability is biologically influenced [14, 21, 22]. Indeed, behaviors that constitute (components of) sociability, such as loneliness and social interaction, are moderately to highly heritable in twin and family studies [23–26]. Genetic association studies of loneliness [27] and social interaction and isolation [28] showed that common genetic variants play a role in these behaviors and that these constructs are genetically associated with depressive symptoms. However, the underlying biological basis of sociability as a construct of multiple behaviors (including loneliness, social interaction, social isolation, and social embarrassment) is still largely undefined as is its genetic link with (brain) disorders.

Psychiatric and neurological disorders that have reduced sociability as a common feature are known to be highly heritable (~60–80% for AD [29], ~64–91% for ASDs [30], ~60% for BPD [31], ~30–40% for MDD [32], and ~79% for SCZ [33]). Genome-wide association studies (GWASs) comparing individuals with these disorders and unaffected controls needed very large sample sizes to robustly identify disease-associated genetic risk variants [34–38]. A limitation of the large case–control GWASs is the probable phenotypic heterogeneity of the cases included; the case-group will contain individuals with very diverse sets of symptoms, severity, and clinical course. Moreover, comorbidity between disorders and overlap in symptomatology between disorders make it difficult to select a group of cases that encompass just one disorder, or a true set of controls that have none. In line with such phenotypic overlap, evidence from twin data [39] as well as recent evidence from the Brainstorm Consortium also documents substantial sharing of common genetic risk factors among psychiatric disorders [40]. The diagnostic distinctions do not seem to align with biological categories, which emphasizes the need for an alternative approach to the investigation of genetic factors involved. Consistent with e.g., Research Domain Criteria (RDoC) approaches [41], investigating the genetics of common traits overlapping between disorders, like reduced sociability, may help increase our understanding of the underlying mechanisms involved in the disorders and their comorbidity.

With sociability being continuously distributed throughout the population, having a biological basis, and reduced sociability being seen in multiple disorders [42, 43], we hypothesized that studying the genetic underpinnings of sociability in the general population can be an alternative way to learn more about the genetics of complex neuropsychiatric disorders. In the current study, we used large cohort data from the UK Biobank (UKBB) [44, 45], in which we computed a sociability score based on the answers to four sociability-related self-report questions. We investigated its genetic architecture, the phenotypic and genetic overlap of the sociability score with AD, ASDs, BPD, MDD, and SCZ, and its link to social behavior in individuals with psychiatric disorders.

## Methods

### Subjects

The UKBB is a major population-based cohort from the United Kingdom that includes individuals aged between 37 and 73 years [44]. The UKBB project was approved by the National Research Ethics Service Committee North West Multi-Center Haydock and all participants provided written informed consent to participate in the study.

### Sociability phenotype

We constructed a sociability measure based on the total score per participant on four questions from the UKBB database that capture different, complementary aspects of sociability: (1) a question about the frequency of friend/family visits, (2) a question on the number and type of social venues that are visited, (3) a question about worrying after social embarrassment, and (4) a question about feeling lonely (see Supplementary Information  1, the sociability score has a range of 0–4). Participants were excluded if they had somatic problems that could be related to social withdrawal (BMI < 15 or BMI > 40, narcolepsy (all the time), stroke, severe tinnitus, deafness or brain-related cancers) or if they answered that they had “No friends/family outside household” (as these persons could not answer the question about the frequency of visits) or “Do not know” or “Prefer not to answer” to any of the questions.

### Phenotypic data on disorders of interest

We grouped individuals with ASDs, MDD, SCZ, BPD, and AD-by-proxy based on the ICD-10 codes provided. For AD-by-proxy, we applied prior definitions from the literature [38], see Supplementary Methods for details. A “not affected” group was created by excluding the above mentioned cases, as well as individuals that fell in the “probable MDD” group (based on [35]), were schizotypical, or manic based on ICD-10 criteria, for details see Supplementary Information 2. Mean values of the sociability score were calculated per group using SPSS 20.0 (SPSS Technologies, Armonk, NY, USA) and compared to “non affected” individuals using general linear models (correcting for age, sex, and assessment center).

### SNP genotyping and quality control

Details about the available genome-wide genotyping data for UKBB participants have been reported previously [44]. Briefly, genotypes were imputed using the Haplotype Reference Consortium, and the UK10K haplotype resource. We accounted for ethnicity and relatedness, see Supplementary Methods for details and excluded individuals with a sex mismatch. Single nucleotide polymorphisms (SNPs) with minor allele frequency <0.005, Hardy–Weinberg equilibrium test *P* value < 1e−6, missing genotype rate >0.05, and imputation quality of INFO <0.8 were excluded. All analyses are based on 342,461 participants of European ancestry for which both genotype data and sociability scores were available.

### Genome-wide association analysis

Genome-wide association analysis with the imputed marker dosages was performed in PLINK2.0, using a linear regression model with the sociability measure as the dependent variable and including sex, age, 10 first PCs, assessment center, and genotype batch as covariates. Robustness analyses were included by running five split-half validation analyses, i.e., splitting our sample five times into two equally sized, randomly selected groups and comparing single-variant results as well as excluding individuals with known psychiatric and neurological disorders based on the ICD-10 codes, see also Supplementary Information 2. Genome-wide association analyses of the four separate questions were performed using linear and linear probability regression models and correcting for sex, age, ten principal components (PCs), assessment center, and genotype batch.

### SNP-based heritability

We applied Linkage disequilibrium (LD) score regression (https://github.com/bulik/ldsc) to estimate the SNP-based heritability from our sociability GWAS summary statistics [46, 47], using precomputed LD scores based on European samples from the 1000 Genomes Project.

### Gene-based analysis

SNP-based *p* values from the main analysis were used as input for the gene-based analysis in MAGMA (v1.07) [48], using all 19,427 protein-coding genes from the NCBI 37.3 gene definitions. We applied a stringent Bonferroni correction to account for multiple testing.

### Genetic correlation analyses

We calculated the bivariate genetic correlation (*r*_g_) between sociability and the publicly available summary statistics of AD, ASDs, BPD, MDD, and SCZ [34, 36, 37, 49, 50] as well as the behavioral traits loneliness, and social anxiety [26, 27] using LD score regression [46, 47].

### Functional annotation and gene-mapping of genomic risk loci

Functional annotation and gene-mapping of genomic risk loci was performed using the Functional Mapping and Annotation (FUMA) online tool, version v1.3.5e (http://fuma.ctglab.nl [51]), including all nominally significant SNPs that were in LD (*r*^2^ ≥ 0.6) with one of the independent genome-wide significant SNPs from the sociability GWAS. Within FUMA, ANNOVAR [52] was used to identify each SNP’s genic position, Combined Annotation Dependent Depletion (CADD) scores [53] were used as a measure of the predicted deleterious effect of a SNP [54] and RegulomeDB scores were used to predict the regulatory functionality of SNPs based on expression quantitative trait loci (eQTLs) and chromatin marks. In addition, the 15-state chromHMM analysis model of epigenomics data from the Roadmap Epigenomics Consortium was used to annotate the minimum predicted chromatin states across tissues for each SNP. Three gene-mapping strategies were used: (1) Positional mapping, based on location (Ensembl v92; GRCh37/hg19), (2) eQTL mapping, based on brain expression data from PsychENCODE, the CommonMind Consortium, BRAINEAC, and GTEx v8 Brain, (3) 3D chromatin interaction mapping, based on chromatin interactions between the SNP region and another gene’s promoter region.

### Enrichment analyses of mapped genes

Through the GENE2FUNC procedure in FUMA (http://fuma.ctglab.nl [51]) we further investigated the set of genes implicated by the gene-mapping approach (described above) in relation to tissue specificity and pathway enrichment. Enrichment analyses of differentially expressed genes (DEGs) were performed across 30 general tissue types and 54 specific tissue types from the GTEx database v8 [55] and across 11 general developmental stages of brain samples and 29 different ages of brain samples from the BrainSpan data [56]. Enrichment of the mapped genes was also assessed using the collection of publicly available predefined gene sets from the Molecular Signatures Database (MsigDB v7.0) [57], WikiPathways (curated version 20191010) [58], and GWAS catalog (version e96 2019-09-24) [59]. FUMA was used with default settings unless stated otherwise. For details see the  Supplementary Methods.

### Polygenic risk score analyses

The PRISM consortium (see https://prism-project.eu/en/prism-study/) collected social behavior-related data as well as genetic data in 6 samples of patient cohorts. The social behavior outcomes used reflect social avoidance, social withdrawal, loneliness, social activities and social relationships. Please see Supplementary Table 1 for the cohorts and social behavior measures that were included and Supplementary Information 3 for details. All PRS analyses were performed in PRSice2 [60]. PRS were calculated for each individual in the independent target samples by scoring the number of risk alleles weighted by their effect in the UKBB sociability GWAS for the set of clumped SNPs using the fast score option in PRSice2. Subsequently, linear regression analyses were performed testing the relationship between the sociability PRS and the social behavior outcomes in the PRISM datasets, including sex, age, and genetic PC’s as covariates, as well as dataset-specific covariates. Multiple comparison correction was performed using Bonferroni correction taking into account the number of tests performed (see Supplementary Information 3).

Further details on all methods performed can be found in the Supplementary Methods.

## Results

### Sociability measure

**Fig. 1:**
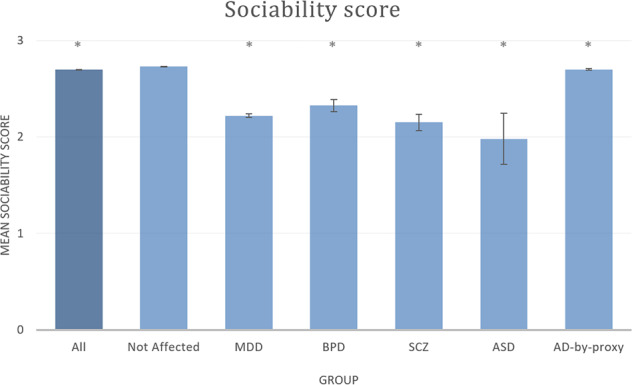
Mean sociability scores in the complete sample (“all”), individuals without psychiatric and neurological disorders (“not affected”) and with specific disorders of interest. AD Alzheimer’s disease, ASD autism spectrum disorders, BPD bipolar disorder, MDD major depressive disorder, SCZ schizophrenia. Error bars show the 95% confidence interval. Groups significantly different from “Not affected” group are indicated with an asterix (*).

Valid data was available for 342,461 adult participants in the UKBB (mean age 56.61 (sd = 8.022), 53.8% female). The range of our sociability score was 0 (low sociability) to 4 (high sociability), with a mean of 2.7 (see Supplementary Information 5). The mean sociability score of participants assigned to one of the psychiatric disorder groups (ASD *n* = 42, BPD *n* = 738, MDD *n* = 8943, and SCZ *n* = 397) was significantly lower than that of the unaffected group. The group of people with an AD-by-proxy status (*n* = 41,648) scored significantly lower than the unaffected group, though the difference with the unaffected group was much less pronounced than for the other disorders (Fig. 1 and Supplementary Table 2).

### Genome-wide association analysis (GWAS)

**Fig. 2:**
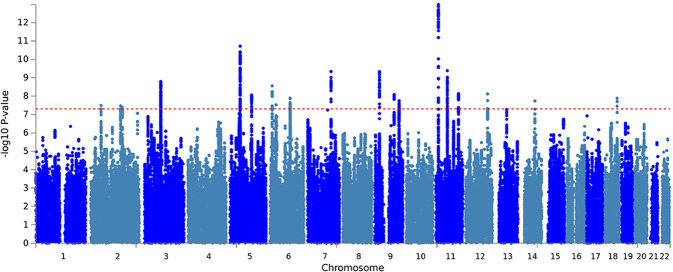
Manhattan plot of the genome-wide association analysis of the sociability score in the UK Biobank sample (*N* = 342,461). Every dot indicates the outcome of the linear regression analysis of one SNP with the sociability measure as the dependent variable and including sex, age, first 10 principal components, assessment center, and genotype batch as covariates. On the *x*-axis the distribution of SNPs over the chromosomes and on the *y*-axis the −log 10 association *p* value is shown. The red dotted line indicates the threshold for genome-wide significance (i.e., *p* = 5e−8).

Our sociability measure had a SNP-based heritability of 0.06 (se = 0.0019). In total, 604 SNPs, with 19 lead-SNPs across 18 independent loci, surpassed the threshold for genome-wide significance (*p* < 5e−8; Table 1, Fig. 2 results are publicly available at 10.17026/dans-ztj-zga6). Split-half validation analyses showed that all variants had robust associations, showing at least nominally significant associations in all five iterations (Supplementary Table 3). Exclusion of individuals with psychiatric and neurological disorders did not change the results substantially, though power was reduced (Supplementary Table 3). Results for the aggregated score were not driven by a single question, as all SNP associations had nominal significance for at least two questions (Supplementary Table 4).

**Table 1:** The location, the significance value, and the nearest gene for the 19 lead-SNPs of the 18 genomic loci that pass the threshold for genome-wide significance (*p* < 5e−8) in the GWAS of the sociability score in the UK Biobank sample.

Lead-SNP	chr	pos	*p* Value	Loci	Start	End	Nearest gene	A1	A2	Beta
rs10180695	2	5,129,2332	3.16E−08	1	51,203,466	51,306,745	*AC007682.1*	T	C	−0.01187
rs202220108	2	148,910,423	3.24E−08	2	148,538,291	148,955,901	*MBD5*	C	A	−0.01194
rs57601252	2	157,100,293	4.00E−08	3	157,009,413	157,150,188	*NR4A2*	A	T	0.01329
rs4266214	3	82,003,207	1.53E−09	4	81,630,790	82,402,319	*RP11-359D24.1*	G	A	−0.01295
rs62365541	5	50,905,846	1.86E−11	5	50,582,954	50,974,050	*CTD-2335O3.3*	A	G	−0.01511
rs4075651	5	107,878,588	8.77E−09	6	107,718,510	107,932,811	*RP11-120B7.1*	C	T	−0.01391
rs10456089	6	11,959,836	2.70E−09	7	11,959,836	12,056,771	*RP11-456H18.1*	A	G	0.02434
rs4839780	6	100,909,398	1.27E−08	8	100,813,469	101,344,679	*SIM1*	C	T	0.01202
rs34979551	6	100,848,871	1.37E−08	8	100,813,469	101,344,679	*SIM1*	G	A	0.01786
rs6976111	7	117,495,667	4.42E−10	9	117,494,829	117,636,111	*CTTNBP2*	A	C	−0.01451
rs3793577	9	23,737,627	4.55E−10	10	23,720,380	23,741,776	*ELAVL2*	A	G	0.01335
rs10761244	9	96,387,592	8.21E−09	11	96,384,524	96,460,883	*PHF2*	C	T	0.01235
rs71507562	9	121,640,304	1.78E−08	12	121,546,458	121,667,198	*TUBB4BP6*	G	T	0.01383
rs34588274	11	13,269,946	1.01E−13	13	13,268,067	13,350,131	*ARNTL*	C	T	−0.01600
rs527528	11	57,433,327	4.02E−10	14	57,404,779	57,756,568	*ZDHHC5*	T	C	−0.01405
rs4245154	11	113,388,674	7.17E−09	15	113,317,745	113,451,229	*DRD2*	G	A	0.01236
rs3742021	12	109,883,117	7.43E−09	16	109,849,297	110,027,795	*MYO1H*	C	T	−0.01335
rs3784060	14	72,114,138	1.73E−08	17	71,757,418	72,162,901	*SIPA1L1*	G	T	0.01315
rs2216270	18	63,656,060	1.25E−08	18	63,650,084	63,668,364	*RP11-389J22.3*	C	T	−0.01540

The nearest gene is based on Ensembl genes [build 85] annotated using ANNOVAR.

*chr* chromosome, *pos* position based on GRCh37/hg19.

In the gene-based genome-wide analysis [48] 56 genes reached significance (Supplementary Table 5). Gene-wide analysis in the nonaffected group showed that the results were not driven by individuals with a diagnosis (Supplementary Table 5).

### Genetic correlation

Our aggregated sociability score showed significant genetic correlations with different components of sociability, i.e., loneliness (*r*_g_ = −0.45, *p* = 2.9e−8), which had been assessed in one of the four questions included in our score, and social anxiety (*r*_g_ = −0.47, *p* = 0.002).

We found highly significant negative genetic correlations between sociability and ASDs (*r*_g_ = −0.27, *p* = 3.6e−28), MDD (*r*_g_ = −0.67, *p* = 4.5e−249), and SCZ (*r*_g_ = −0.15, *p* = 7.5e−23). No significant genetic correlations were found between sociability and BPD or AD (Table 2).

**Table 2:** Genetic correlation between the sociability score in UK Biobank (Soc) and autism spectrum disorders (ASD), major depressive disorder (MDD), schizophrenia (SCZ), bipolar disorder (BP), and Alzheimer’s disease (AD), and quantitative measures of Loneliness and Social Anxiety (SocAnx).

Comparison	Rg (se)	*p* Value	Sample size	PMID
**Soc-Loneliness**	**−0.4518 (0.0815)**	**2.98E−08**	**7556**	**27629369**
**Soc-SocAnx**	**−0.4743 (0.154)**	**2.10E−03**	**11,268**	**28224735**
Soc-AD	−0.039 (0.028)	5.74E−01	17,008 cases, 37,154 controls	24162737
**Soc-ASD**	**−0.2692 (0.0245)**	**3.60E−28**	**18,381 cases, 27,969 controls**	**30804558**
Soc-BPD	−0.0143 (0.019)	4.50E−01	20,352 cases, 31,358 controls	31043756
**Soc-MDD**	**−0.6746 (0.02)**	**4.53E−249**	**59,851 cases, 113,154 controls**	**29700475**
**Soc-SCZ**	**−0.1532 (0.0156)**	**7.50E−23**	**34,241 cases, 45,604 controls, 1235 trios**	**25056061**

Significant genetic correlations are indicated in bold.

### Polygenic risk score (PRS) analyses

PRS analysis in six patient-only samples of BPD, MDD, and SCZ cases (427 ≤ *n* ≤ 1705) showed an association between polygenic load for sociability and “interpersonal relations” in BPD patients and two additional nominally significant associations (Supplementary Table 6).

### Functional follow-up

Among the 1953 candidate SNPs, there was an enrichment for intergenic SNPs (55.6%; enrichment 1.19; *p* = 2.63e−15) compared to the reference panel, while SNPs in intronic (30.2%; enrichment 0.83; *p* = 8.94e−09), exonic (0.1%; enrichment 0.10; *p* = 1.14e−06), and noncoding RNA (0.5%; enrichment 0.45; *p* = 9.37e−03) regions were significantly underrepresented (Supplementary Fig. S1). Two SNPs were located in exons of protein-coding genes, namely rs2303751 in *ISL1* and rs2229519 in *GBE1*; the latter had a CADD-score of 23.9. Overall, 104 SNPs (5.3%) had CADD-scores >12.37 (i.e., are potentially pathogenic), and these were distributed across 16 of the 18 significant genomic loci. Analysis of potential regulatory functions of SNPs using RegulomeDB, where data was available for 1515 candidate SNPs, showed that 23 (1.5%) had a score >2, indicating they were highly likely to have regulatory functions; more generally, the majority of the annotated candidate SNPs (*N* = 1391; 71.2%) were located in open chromatin regions (Supplementary Fig. S2). Detailed results on annotation of candidate SNPs can be found in Supplementary Table 7.

Gene-mapping identified 76 genes likely implicated in sociability genetics. Positional gene-mapping provided support for 31 genes (40.8%), eQTL-mapping implicated 25 genes (32.9%), and 3D chromatin mapping implicated 47 genes (61.8%). Across approaches, 23 genes (30.3%) were implicated by at least two approa ches and 4 genes (*HIVEP1, MED19*, *TMX2*, and *DRD2)* were identified by all strategies. Compared to the MAGMA gene-based analysis 18 genes were overlapping. Detailed gene-mapping results are given in Supplementary Table 8, and schematic representations of the chromatin interaction results are shown in Supplementary Fig. S3. Follow-up analyses were performed to examine tissue expression enrichment and pathway enrichment of the 76 FUMA-mapped genes. Analyses with GTEx data [55] (30 general tissue types and 54 specific tissue types) and with BrainSpan data [56] (11 general developmental stages of brain samples and 29 different ages of brain samples) identified no significantly enriched differentially expressed gene sets (Supplementary Fig. S4). Gene-set enrichment analyses identified seven significantly enriched positional gene sets (MsigBD c1) [57], one significantly enriched gene set from WikiPathways [58] (i.e., the “*Amino acid conjugation of benzoic acid*” pathway), and significant enrichment among the set of 19 GWAS catalog reported genes [59]. The significantly enriched gene sets are shown in Supplementary Fig. S5.

## Discussion

In this study, we showed that sociability, scored based on answers to four social functioning-related self-report questions in the general population sample of UKBB, was decreased by 15–27% in individuals with ASDs, MDD, SCZ, and BPD, but only by 1% in individuals with AD (by-proxy). This sociability construct showed moderate genetic correlation with loneliness and social anxiety that encompass more specific, single aspects of sociability. Consistent with the phenotypic overlap observed, the sociability score was also negatively genetically correlated with ASDs, MDD, and SCZ. The phenotypic overlap of sociability with BPD and AD was not accompanied by genetic correlations. Through SNP-based and gene-based GWAS, we identified 18 independent loci and 56 genes associated with the sociability score. Using functional analysis we found that several SNPs are thought to have deleterious effects on the gene products.

This is the first study to investigate the genetics of sociability as an aggregated construct, including aspects of loneliness, social relationships, social embarrassment, and social activities. Although other studies have investigated the influence of common genetic variants on single aspects of this construct, like loneliness [27, 61], or related ones, like social anxiety [26], we aimed to capture a broader concept of sociability. Although the four items only correlate with each other to a limited extend, we have selected them to reflect four different, complementary aspects of sociability, including quantitative behavioral aspects of sociability (question 1), qualitative aspects of sociability (question 2) and subjective aspects of sociability that are linked to social anxiety/avoidance (question 3 and 4). Based on the phenotypic associations of our aggregated score to multiple psychiatric disorders we believe our construct captures a cross-disorder trait and the evidence that our genetic signals are not driven by single questions indicates the added value of our combined score. A previous study in UKBB performed a multi-trait (MTAG) design study of social interaction, including loneliness, frequency of social interactions, and ability to confide in someone [28]. We decided to use an additive construct, preferring this over the multi-trait design because our data did not satisfy the assumptions on, amongst others, the extent of the genetic correlation values required for performing MTAG analyses [62]. Our aggregated measure of different aspects of sociability was influenced significantly by common genetic variants. The SNP-based heritability of the score was 6%, which was nominally higher than the SNP-based heritability reported for the multi-trait study of social interactions (*h*^2^ = 3.4–5%; [28], but lower than the heritability observed for more restricted aspects of sociability, i.e., loneliness (*h*^2^ = 4.2–16%; [27, 28], and social anxiety (*h*^2^ = 12%; [26]).

We were able to show that the sociability score constructed in the general population sample picked up aspects of social behavior altered in different brain disorders. For individuals with ASDs, MDD, and/or SCZ, we found 19% or more reductions in the mean of the sociability score compared to the unaffected group. In line with this, we also showed that sociability was genetically correlated with ASDs, MDD, and SCZ, with a particularly strong negative correlation for MDD. As a part of the IMI2 consortium PRISM, we investigated genetic aspects of sociability and the active process of social withdrawal using patient cohorts from consortium partners. While power was limited, we did see nominal associations with a sociability score PRS for several of the phenotypic measures, providing additional evidence for the relevance of population sociability scores for social functioning of patients with psychiatric disorders. We were limited in these studies by the fact that the different cohorts had used different instruments to measure aspects of sociability.

For BPD, findings were different than for the other psychiatric disorders investigated. Distinctions between BPD and the other disorders are also seen more often, both phenotypically, with for example higher premorbid adjustments in BPD compared to SCZ [63], as well as genetically, with for example positive genetic correlations between educational attainment and BPD, while educational attainment is negatively correlated to MDD [64] and SCZ [65]. Patients with BPD fluctuate between depressive and manic states. Reduced sociability is pronounced in depressive periods, but also observed during remission phases [66]. While manic episodes are often characterized by an increase in social visits, feeling less socially embarrassed, and having the energy to visit public social places, the aspects covered in our sociability score. After finding the reduced sociability score in BPD individuals, we were somewhat surprised not to find any genetic correlation for the sociability score and BPD risk. This may be explained by the known (genetic) heterogeneity of BPD [37] in combination with the more limited extent of social deficits in this disorder. Alternatively, reduced sociability in BPD patients might rather be explained by environmental risk factors that are shared or because of unexplained genetic variance in both traits and we cannot rule out power issues in the discovery GWAS used to calculate the correlation.

Reduced sociability has previously been suggested as a risk factor and early behavioral sign of AD [67, 68], and linked with cortical amyloid burden, a putative biomarker of AD [69]. We only found a minimal (1%) reduction in our sociability score in AD-by-proxy individuals, which included both diagnosis and family history of AD, and has been reported to capture AD risk in previous UKBB genetic studies [38, 70]. Similarly, we did not find significant genetic correlations between the sociability score and AD risk. Different explanations might capture this discrepancy. The few AD patients in UKBB may represent a group biased towards better cognitive/social performance, or the AD by-proxy group may not carry many genetic risk factors for social deficits, or may only develop those deficits at a later stage. Alternatively, the aspects of social behavior included in our sociability score may not optimally capture the social deficits relevant to AD. With regard to the lack of genetic overlap, one may consider that the social deficits in AD may not be genetic in nature but rather occur downstream of environmental risk factors and we cannot rule out power issues in the discovery GWAS used to calculate the correlation. The apparent discriminatory power of our sociability score for psychiatric disorders and AD may form an interesting starting point for follow-up research.

By performing a GWAS of the sociability score, we were able to detect 18 robust genome-wide significant independent loci, including 19 lead-SNPs. Functional analysis in FUMA indicated that that several SNPs are thought to have deleterious effects on the gene products. While this may suggest an increase in the plausibility of their implication on a given disease or trait, this is by no means determinant and caution is highly recommended to avoid over-interpretation. Further studies are necessary to determine whether or not they drive the association at a given locus.

The strongest association signal in the SNP-based GWAS was observed on chromosome 11p15 (rs34588274, *p* = 1.01e−13). This locus encompasses the *ARNTL* gene, which is a circadian clock gene also known as BMAL1 [71]. Circadian disruptions, like delayed sleep, reduced sleep efficiency, difficulties falling asleep, early waking, and higher levels of day-time sleepiness, are common features of MDD, SCZ, BPD, and several neurodegenerative diseases [72–74]. Genetic variants within clock genes, including *ARNTL*, have been previously associated to psychiatric disorders and AD [75–77]. Interestingly, the lead-SNP in this region, rs34588274, has previously been associated with neuroticism, the well-being spectrum (life satisfaction, positive affect, neuroticism, and depressive symptoms), and BMI (GWAS catalog, https://www.ebi.ac.uk/gwas/ [78, 79]). Another significant locus worth highlighting is located on 11q22 (rs4245154, *p* = 7.17E−09); it includes *DRD2*, a gene that has been extensively studied in multiple disorders, including SCZ. This gene encodes the D2 subtype of the dopamine receptor that is the target of all currently used antipsychotics [80, 81]. *DRD2* is one of the few candidate genes for SCZ, that have been confirmed by GWAS [36]. Our top-SNP for the gene was recently also found to be significantly associated with MDD [35, 36]. Importantly, the association with *DRD2* remained significant when we excluded neuropsychiatric cases from our sociability analyses, indicating that the association is not driven by diagnosed individuals in our sample. A third interesting genome-wide significant locus was located on chromosome 9p21 (rs3793577, *p* = 4.55E−10). *ELAVL2* has also been shown to be associated with MDD in the latest GWAS [35]. The protein encoded by this gene is a neuron-specific RNA-binding protein that is involved in several aspects of neuronal functioning important for normal functioning of the brain [82]. Co-expression networks for *ELAVL2* highlight its connection to neurodevelopmental genes, implying it to be a potential important gene for neurodevelopmental disorders more general.

This study should be viewed in light of some strengths and limitations. A major strength of the current study is the large sample size, providing us with the power to detect single-variant genetic associations. Another main strength is the combined phenotype that is capturing a sociability construct shared between disorders. A limitation of the current study is the modest SNP-based heritability, indicating we are only touching on part of the heritability of the complex multifactorial disorders showing reduced sociability as an overlapping trait. Another limitation is that our sociability questionnaire addresses average behaviors over a time frame of maximally 1 year and does not take into account sociability-related behaviors that may be present or absent in specific time frames, e.g., during manic episodes in BPD. Further, as the current study has been conducted in a cohort of European-only participants, this limits the generalizability of our results across other ancestral populations, and the field is in current need of more genetic diversity and future work is needed to address this.

In conclusion, our data shows that there is a significant genetic component to the variation in (population) levels of sociability. This genetic contribution to sociability is relevant to the psychiatric disorders ASDs, MDD, and SCZ, but not to BPD and the neurological disorder AD.

## Funding and disclosure

The PRISM project (www.prism-project.eu) leading to this work has received funding from the Innovative Medicines Initiative 2 Joint Undertaking under grant agreement No 115916. This Joint Undertaking receives support from the European Union’s Horizon 2020 research and innovation program and EFPIA. This work reflects only the authors’ views, and neither IMI JU nor EFPIA nor the European Commission are liable for any use that may be made of the information contained therein. Further support was provided by the EU H2020 Program under the Innovative Medicines Initiative 2 Joint Undertaking with grant agreement 777394 (AIMS-2-TRIALS), the Spanish Ministry of Science, Innovation and Universities, Instituto de Salud Carlos III (PI14/00397, PI14/02103, PIE16/00055, PI17/00819, PI17/00481), co-financed by ERDF Funds from the European Commission, “A way of making Europe”, CIBERSAM, Madrid Regional Government (B2017/BMD-3740 AGES-CM-2), EU Structural Funds, EU Seventh Framework Program under grant agreement FP7-HEALTH-2013-2.2.1-2-603196 (Project PSYSCAN), Fundación Familia Alonso, Fundación Alicia Koplowitz. This work is also part of the research program *Computing Time National Computing Facilities Processing Round pilots 2018* with project number *17666*, which is (partly) financed by the Dutch Research Council (NWO). This work was carried out on the Dutch national e-infrastructure with the support of SURF Cooperative. CA has been a consultant to or has received honoraria or grants from Acadia, Angelini, Gedeon Richter, Janssen Cilag, Lundbeck, Otsuka, Roche, Sage, Servier, Shire, Schering Plough, Sumitomo Dainippon Pharma, Sunovion, and Takeda. NvdW received speaking bureau honoraria from Eli Lilly and Wyeth and served on advisory panels of Eli Lilly, Pfizer, Wyeth, and Servier. BP has received (non-related) research grants from Jansen research and Boehringer Ingelheim. AS is or has been consultant/speaker for: Abbott, Abbvie, Angelini, Astra Zeneca, Clinical Data, Boheringer, Bristol Myers Squibb, Eli Lilly, GlaxoSmithKline, Innovapharma, Italfarmaco, Janssen, Lundbeck, Naurex, Pfizer, Polifarma, Sanofi, and Servier. BF has received educational speaking fees from Medice. GP is director of Drug Target ID, Ltd. The authors declare no competing interests.

## Supplementary information

Supplementary files

Supplementary Table 7

Supplementary Table 8
