# Supplementary material for: Genetic underpinnings of sociability in the general population
Source: Neuropsychopharmacology. 2021 May 30;46(9):1627–34. doi: 10.1038/s41386-021-01044-z (PMC8280100; doi:10.1038/s41386-021-01044-z)
Supplement: Supplementary file 1 — Supplementary files [file 41386_2021_1044_MOESM1_ESM.pdf]

SUPPLEMENTARY DATA

Supplementary Methods

Subjects

The UK Biobank (UKBB) is a major population-based cohort from the United Kingdom that includes more than 500,000 individuals aged between 37 and 73 years. The initial data was collected between 2006 and 2010 and includes questionnaire data, genetic data and neuroimaging data. The study design and sample characteristics of the UKBB (http://www.ukbiobank.ac.uk/about-biobankuk/) have been extensively described elsewhere [1,2]. The UKBB project was approved by the National Research Ethics Service Committee North West Multi-Centre Haydock and all participants provided written informed consent to participate in the study.

Sociability phenotype

Four questions were selected from the UKBB database based on their link to sociability: 1. How often do you visit friends or family or have them visit you? ; 2. Which of the following do you attend once a week or more often? (selection from: sports club or gym, pub or social club, religious group, adult educational class, other group activity) ; 3. Do you worry too long after an embarrassing experience? ; 4. Do you often feel lonely?. Based on the answers to these four questions, we constructed a sociability measure for which the total score per participant is the sum of the scores on the 4 questions (see **Supplement 1**). Participants were excluded if they had somatic problems that could be related to social withdrawal (BMI < 15 or BMI > 40, narcolepsy (all the time), stroke, severe tinnitus, deafness or brain-related cancers) or if they answered that they had “No friends/family outside household” to the question ‘How often do you visit friends or family or have them visit you?’ or “Do not know” or “Prefer not to answer” to any of the questions. The sociability score ranged from 0-4.

Phenotypic data on disorders of interest

We grouped individuals with psychiatric and neurological disorders of interest within the UKBB cohort. For ASDs, individuals were grouped that had childhood autism, atypical autism, or Asperger's syndrome (based on ICD 10-codes). For MDD, individuals were grouped according to ICD 10-codes and using the selection criteria from Howard et al., 2018[3]. For SCZ and BPD, individuals were grouped that had an ICD 10 code-based diagnosis of schizophrenia and bipolar disorder, respectively. Lastly, for AD, individuals were grouped by combining the Alzheimer's diagnosis ICD 10 code with Alzheimer's family history based on their parents. In this way, and based on the approach by Jansen et al., 2019[4], all participants were included that may develop AD but had not reached the age of onset at the time they were assessed, creating an 'AD-by-proxy' measure. Further, a "not affected" group was created by excluding the above mentioned ASD, MDD, SCZ, BPD, and AD cases, as well as individuals that fell in the 'probable MDD' group (based on [5]), were schizotypical, or manic based on ICD10 criteria, for details see the **Supplement 5** in the Supplementary data. Mean values of the sociability score and distributions were calculated per group using SPSS 20.0 (SPSS Technologies, Armonk, NY, USA) and compared to 'non affected' individuals using general linear models (correcting for age, sex, and assessment center).

SNP genotyping and quality control

Details about the available genome-wide genotyping data for UKBB participants have been reported previously [1]. We used third-release genotyping data (see https://biobank.ctsu.ox.ac.uk/crystal/label.cgi?id=100319). Briefly, 49,950 participants were genotyped using the UK BiLEVE Axiom Array and 438,427 participants were genotyped using UK Biobank Axiom Array. Genotypes were imputed into the dataset using the Haplotype Reference Consortium (HRC), and the UK10K haplotype resource. To account for ethnicity, we included only those individuals that identified themselves as "white" by self-report and plotted the Principal Components (PC) provided by the UKBB, excluding individuals considered to be outliers according to PCs 1 and 2. Genetic relatedness calculated with KING kinship and provided by the UKBB

(https://kenhanscombe.github.io/ukbtools/articles/explore-ukb-data.html ; http://www.ukbiobank.ac.uk/wp-content/uploads/2014/04/UKBiobank_genotyping_QC_documentation-web.pdf) was used to identify first and second-degree relatives. Subsequently ´families´ (i.e. clusters of related individuals above an IBD>0.125 threshold) were created and only one individual from each of these created 'families' was included in the analysis. If self-reported sex and SNP-based sex differed, individuals were excluded from further analysis. Single nucleotide polymorphisms (SNPs) with minor allele frequency <0.005, Hardy-Weinberg equilibrium test P value<1e−6, missing genotype rate >0.05, and imputation quality of INFO <0.8 were excluded. In the current study, all analyses are based on 342,461 participants of European ancestry for which both genotype data and sociability scores were available.

Genome-wide association analysis

Genome-wide association analysis with the imputed marker dosages was performed in PLINK2.0, using a linear regression model with the sociability measure as the dependent variable and including sex, age, 10 first PCs, assessment center, and genotype batch as covariates. SNPs were considered significantly associated if they had p-value < 5e-8. Associated loci were considered independent of each other at r^2 0.6 and lead SNPs were classified as the SNP with the smallest association p-value and at r^2 0.1, using a 250kb window. Robustness analyses were included by running five split-half validation analyses, i.e. splitting our sample five times in two equally sized, randomly selected groups and comparing single variant results as well as excluding individuals with known psychiatric and neurological disorders (based on the selection we made in '*Phenotypic data on disorders of interest*'). Genome-wide association analyses of the four separate questions that together comprise our sociability score were also performed using linear and linear probability regression models and correcting for sex, age, 10 PCs, assessment center, and genotype batch.

SNP-based heritability

To assess the proportion of phenotypic variance explained by common genetic variants, we applied Linkage disequilibrium (LD) score regression (https://github.com/bulik/ldsc) that estimates the heritability attributable to genome-wide SNPs (SNP-based heritability) from our sociability GWAS summary statistics based on the slope of the LD score regression [6,7]. We used pre-computed LD scores based on European samples from the 1000 Genomes Project, as indicated in https://github.com/bulik/ldsc/wiki/Heritability-and-Genetic-Correlation.

Gene-based analysis

Apart from the SNP-based GWAS, we conducted a gene-based association analysis. For this, SNP-based p-values from the main analysis were used as input for the gene-based analysis in MAGMA (v1.07) [8], using all 19,427 protein-coding genes from the NCBI 37.3 gene definitions. Gene-based p-values were then calculated using the SNP-wise mean model (default for summary statistics analysis) that combines the effects of SNPs within a gene and uses the sum of –log(SNP P-values) as test statistic. In order to account for linkage disequilibrium (LD) between the SNPs, we used the European dataset of the 1000 Genomes Phase 3 as reference, provided at MAGMA's website (https://ctg.cncr.nl/software/MAGMA/ref_data/g1000_eur.zip). After SNP annotation, there were 18,186 genes that were covered by at least one SNP. We applied a stringent Bonferroni correction to account for multiple testing through adjusting for the number of genes tested, setting the genome-wide gene-based threshold for significance at $p<2.75e-6$.

Genetic correlation analyses

To evaluate the extent of shared common variant-based genetic architectures between sociability and AD, ASDs, BPD, MDD, and SCZ [9-13] as well as the behavioral traits loneliness, and social anxiety [14,15], the bivariate genetic correlations attributable to genome-wide SNPs (r_g) were calculated using LD score regression. The GWAS summary statistics underwent additional filtering steps. Only markers

overlapping with HapMap Project Phase 3 SNPs and passing the INFO score ≥0.9 and MAF ≥0.01 filters were included (when information on INFO scores and MAF were available). SNPs with missing values, duplicate rs-numbers, too low sample size (when available SNPs with an effective sample size less than 0.67 times the 90th percentile of the sample size), or that were strand-ambiguous - as well as indels – were removed. We used precalculated LD scores ('eur_w_ld_chr/' files; (see https://github.com/bulik/ldsc [6,7,16]) for each SNP using individuals of European ancestry from the 1000 Genomes project that are suitable for LD score analysis in European populations.

Functional annotation of genomic risk loci

Functional annotation of genomic risk loci was performed using the Functional Mapping and Annotation (FUMA) online tool, version v1.3.5e (http://fuma.ctglab.nl [17]). Annotation of candidate SNPs was carried out for all nominally significant SNPs that were in LD (r2>=0.6) with one of the independent genome-wide significant SNPs from the sociability GWAS. Using the European data from the 1000 Genomes Project (Phase 3) as reference, FUMA may identify and annotate additional candidate SNPs that are in strong LD with the independent significant SNPs but that were not present in the GWAS data. Within FUMA, ANNOVAR [18] was used to identify each SNP's genic position (e.g. intronic, exonic, etc.). Combined Annotation Dependent Depletion (CADD) scores [19] were used as a measure of the predicted deleterious effect of a SNP on protein structure/function (i.e., SNPs with CADD >12.37 are considered potentially pathogenic [20]). We also used RegulomeDB scores [21] that represent predicted regulatory functionality of SNPs based on expression quantitative trait loci (eQTLs) and chromatin marks and that range from 1a to 7, with lower scores indicating SNPs most likely to have regulatory function. In addition, the 15-state chromHMM analysis model of epigenomics data from the Roadmap Epigenomics Consortium[22] was used to annotate the minimum predicted chromatin states across tissues for each SNP, where states 1-7 refer to open chromatin states.

Gene-mapping

SNPs within genome-wide significant loci in the sociability GWAS were mapped to genes using the FUMA online tool (v1.3.5e[17]). Three gene-mapping strategies were used: (1) Positional mapping, SNPs were mapped to genes if they were located within a 10kb window from known protein-coding genes in the human reference panel (Ensembl v92; GRCh37/hg19); (2) eQTL mapping, SNPs were mapped to genes (up to 1Mb apart) if they were associated with the expression level of that gene, based on brain expression data from PsychENCODE [23], the CommonMind Consortium [24], BRAINEAC [25], and GTEx v8 Brain [26]. A false discovery rate of 0.05 was used to identify significant SNP-gene pairs; (3) 3D chromatin interaction mapping, SNPs were mapped to genes if there was evidence of significant chromatin interactions between the SNP region and another gene's promoter region (i.e. 250bp upstream and 50bp downstream of the gene's transcription start site). Mapping was based on FUMA's built-in chromatin interaction data from PsychENCODE (EP links and Promoter anchored loops)[23], Hi-C data from adult cortex and fetal cortex [27], and Hi-C data specifically for dorsolateral prefrontal cortex and hippocampus [28]. Enhancer and promoter regions were annotated based on the 12 brain-related epigenomes from the Roadmap Epigenome Project [22].

Polygenic risk score analyses

Samples: The PRISM consortium (see https://prism-project.eu/en/prism-study/) collected social behavior-related data as well as genetic data in 6 samples of patient cohorts. Please see **Supplementary Table 1** for the cohorts and social behavior measures that were included and **Supplement 3** for details.

Analysis: All PRS analyses were performed in PRSice2[29]. LD-clumping was run to obtain a relatively independent set of SNPs, while keeping the most significant SNPs in each LD block (r2=0.1, window=250kb). PRS were calculated for each individual in the independent target samples by scoring

the number of risk alleles weighted by their effect as estimated through the beta values in the UKBB sociability GWAS for the set of clumped SNPs using the fast score option in PRSice2. Subsequently, linear regression analyses were performed testing the relationship between sociability PRS and the social behavior outcomes in the PRISM datasets, including sex, age and genetic PC's as covariates, as well as dataset-specific covariates. Multiple comparison correction was performed using Bonferroni correction taking into account the number of test performed (see **Supplement 3**).

Supplement 1: Scoring of the questions

Total score for sociability=sum of scores on questions 1-4; range: 0-4.

<u>QUESTIONS and SCORING</u>

1. Frequency of friend / family visits (Data-Field 1031)

How often do you visit friends or family or have them visit you? (Hint: If this varies, please give an average of how often you visit or have had visits in the last year. Include meeting with friends or family in environments outside of the home such as in the park, at a sports field, at a restaurant or pub.)

ANSWER	***SCORE***
1. Almost daily	*1*
2. 2-4 times a week	*0.8*
3. About once a week	*0.6*
4. About once a month	*0.4*
5. Once every few months	*0.2*
6. Never or almost never	*0*
7. No friends/family outside household	*- exclude*
8. Do not know	*- exclude*
9. Prefer not to answer	*- exclude*

2. Leisure / social activities (Data-Field 6160)
Which of the following do you attend once a week or more often? (You can select more than one) (Hint: If this varies, please think about activities in the last year.)

ANSWER	*SCORE*
1. Sports club or gym	*At least one of these answers: 1*
2. Pub or social club	
3. Religious group	
4. Adult education class	
5. Other group activity	
6. None of the above	*0*
7. Prefer not to answer	*- exclude*

3. Worry after social embarrassment (Data-Field 2000)
Do you worry too long after an embarrassing experience?

ANSWER	*SCORE*
1. Yes	*0*
2. No	*1*
3. Do not know	*- exclude*
4. Prefer not to answer	*- exclude*

4. Loneliness (Data-Field 2020)
Do you often feel lonely?

ANSWER	*SCORE*

1. Yes	*0*
2. No	*1*
3. Do not know	*- exclude*
4. Prefer not to answer	*- exclude*

Supplement 2

UKBB datafields used for ICD coding

SCZ: F200, F201, F202, F203, F204, F205, F206, F207, F208, F209
MDD: F320, F321, F322, F323, F328, F329, F330, F331, F332, F333, F334, F338, F339, F340, F341, F348, F380, F388, F39
BP:F310, F311, F312, F313, F314, F315, F316, F317, F318, F319
ASD: F84
Manic: F30
Schizotypy: F21, F22, F23, F24, F25, F28, F29
The probable MDD phenotype was derived based on the description in Howard et al. Based on questions concerning "depressed/down for a whole week (UK Biobank field: 4598); plus at least 2 weeks duration (UK Biobank field: 4609); plus ever seen a GP or psychiatrist for nerves, anxiety or depression" (UK Biobank fields: 2090 and 2010), or "ever anhedonia for a whole week (UK Biobank field: 4631); plus at least 2 weeks duration (UK Biobank field: 5375); plus ever seen a GP or psychiatrist for nerves, anxiety, or depression" (UK Biobank fields: 2090 and 2010).

Supplement 3: Description of the PRISM cohorts & PRS analyses

NESDA:

The Netherlands Study of Depression and Anxiety (NESDA)[30] includes patients diagnosed (Diagnostic and Statistical Manual of Mental Disorders-IV) with a lifetime diagnosis of an anxiety or depressive disorder, established with the use of the Composite Interview Diagnostic Instrument (CIDI) – lifetime version 2.1. Patients were excluded if they had a primary diagnosis of bipolar, psychotic, obsessive compulsive or severe addictive disorder and when participants did not speak fluently Dutch. Participants were genotyped using the Perlegen-Affymetrix 5.0 en Affymetrix 6.0. Genotypes were imputed into the dataset using the Haplotype Reference Consortium (HRC) resource. Single nucleotide polymorphisms (SNPs) with minor allele frequency <0.01, and imputation quality of INFO < 0.3 were excluded. PRS analyses were performed using the UKBB sociability GWAS as base and the De Jong Gierveld loneliness scale as a target. Covariates that were included in the analyses are sex, age and 10 PCs. The sample used in the analysis included 1705 individuals, of which 1162 were females, and the mean age was 43.2 years.

GRSD:

This sample was recruited by the European Group for the Study of Resistant Depression (GSRD) and included patients had a diagnosis of major depressive disorder (MDD), for details see [31]. Participants were genotyped using the Illumina Infinium PsychArray 24 BeadChip (Illumina, Inc., San Diego). Genotypes were imputed using the Haplotype Reference Consortium (HRC r1.1 2016). Single

nucleotide polymorphisms (SNPs) with minor allele frequency < 0.01 and imputation quality of R2 < 0.3 were excluded. PRS analyses were performed using the UKBB sociability GWAS as base and the Items 2 and 3 (social and family functioning) of the Sheehan scale as a target. Covariates that were included in the analyses are sex, age, center of recruitment, 10 population PCs, and general psychopathological severity (total Montgomery-Åsberg Depression Rating Scale (MADRS)).

STAR*D:

The Sequenced Treatment Alternatives to Relieve Depression (STAR*D) study included patients with major depressive disorder (MDD). Genome-wide data available in STAR*D were obtained using Affymetrix Human Mapping 500 K Array Set or Affymetrix Genome-Wide Human SNP Array 5.0 (Affymetrix, South San Francisco, California), for details see [32]. Genotypes were imputed into the dataset using the Haplotype Reference Consortium (HRC r1.1 2016). Single nucleotide polymorphisms (SNPs) with minor allele frequency <0.01, and imputation quality of R2 < 0.3 were excluded. PRS analyses were performed using the UKBB sociability GWAS as base and Work and social Adjustment Scale (WSAS) Items 3 and 5 (social activities and relationships) as target. Covariates that were included in the analyses are sex, age, center of recruitment, 10 population PCs, general psychopathological severity (Quick Inventory of Depressive Symptomatology clinician-rated (QIDS-C).

CATIE:

The CATIE study included patients with schizophrenia (SCZ). Genome-wide data available in CATIE were obtained using Affymetrix Human Mapping 500 K Array Set or Affymetrix Genome-Wide Human SNP Array 5.0 (Affymetrix, South San Francisco, California), for details see [33]. Genotypes were imputed into the dataset using the Haplotype Reference Consortium (HRC r1.1 2016). Single nucleotide polymorphisms (SNPs) with minor allele frequency <0.01, and imputation quality of R2 < 0.3 were excluded. PRS analyses were performed using the UKBB sociability GWAS as base and items n4 (passive/apathetic social withdrawal) and g16 (active social avoidance) of the Positive and Negative Syndrome Scale (PANSS) as target. Covariates that were included in the analyses are sex, age, center of recruitment, 4 population PCs, general psychopathological severity (PANSS score minus the items used for creating the phenotype).

STEP-BD:

The Systematic Treatment Enhancement Program for Bipolar Disorder (STEP-BD) study included patients with Bipolar Disorder (BD). Genome-wide data available in STEP-BD were obtained using Affymetrix Gene Chip Human Mapping 500 K Array Set (Affymetrix, South San Francisco, California), for details see [34]. Genotypes were imputed into the dataset using the Haplotype Reference Consortium (HRC r1.1 2016). Single nucleotide polymorphisms (SNPs) with minor allele frequency <0.01, and imputation quality of R2 < 0.3 were excluded. PRS analyses were performed using the UKBB sociability GWAS as base The Range of Impaired Functional Tool (LRIFT) items 2c (interpersonal relations with relatives) and 2d (interpersonal relations with friends) as target. Covariates that were included in the analyses are sex, age, center of recruitment, 4 population PCs, general psychopathological severity (MADRS and Young Mania Rating Scale (YMRS).

Description of phenotypic distribution of the samples is below (before standardization and correction for covariates; for social withdrawal, mean (SD) and range are reported). Correlation is referred to the Pearson’s correlation coefficient.

Sample	Social withdrawal	Symptom severity	Correlation between social withdrawal and total severity	Age	Gender (F/M)
STAR*D	9.86 (3.99)<br>0-16	16.06 (3.31)	0.40 (0.35-0.45)	43.66 (13.48)	695/451
GSRD	12.67 (5.03)<br>0-20	24.75 (11.32)	0.60 (0.56-0.64)	51.82 (13.86)	734/384
CATIE	6.09 (2.46)<br>2-13	67.47 (16.29)	0.53 (0.47-0.59)	41.11 (11.50)	109/369
STEP-BD	4.71 (1.84)<br>2-10	MADRS: 15.96 (11.29)<br>YMRS: 6.42 (6.59)	MADRS: 0.40 (0.33-0.46)<br>YMRS: 0.15 (0.08-0.23)	40.67 (12.33)	412/309

ROM study

The population recruited for this study came from the Routine Outcome Monitoring (ROM) study. These participants were ambulatory out-patients that had partaken in an extensive ROM interview between 2007 and 2011 as part of routine patient care. Detailed ROM protocol information has previously been published elsewhere [35]. A total of 949 participants collected saliva samples for the Mood, Anxiety, and Somatoform disorders and HPA-axis biobank (MASHbank), using a protocol that was similar to the protocol used in the Netherlands Study of Depression and Anxiety (NESDA, [36]). The protocol was approved by the LUMC Ethical Review Board. Participants gave their written consent to partake in the study, and the study was carried out in accordance with the principles of the declaration of Helsinki. Of these participants, 489 had completed the Dimensional Assessment of Personality Pathology-Short Form (DAPP-SF) social avoidance subscale (consisting of 6 items)[37], the Brief Symptom Inventory (BSI) social withdrawal subscale (consisting of 5 items[38]), and the Short Form-36 social functioning subscale (consisting of 2 items)[39]. Genome-wide data available in the ROM study were obtained using the PsychChip platform of Affymetrix (Affymetrix, South San Francisco, California) Genotypes were imputed into the dataset using the Haplotype Reference Consortium (HRC). Single nucleotide polymorphisms (SNPs) with minor allele frequency <0.01, and imputation quality of INFO < 0.3 were excluded. Covariates that were included in the PRS analyses are sex, age, and 10 population PCs.

Supplement 4: Correlations between the four individual questions

	Q1	Q2	Q3	Q4
Q1	1			
Q2	0.026	1		
Q3	0.051	0.191	1	
Q4	0.079	0.018	0.037	1

Note: Pearson correlations between the four individual questions. Q1: frequency of friend/family visits, Q2:Worry too long after embarrassement, Q3: Loneliness, Q4: Leisure/social activities

Supplement 5: Distribution of total sociability score

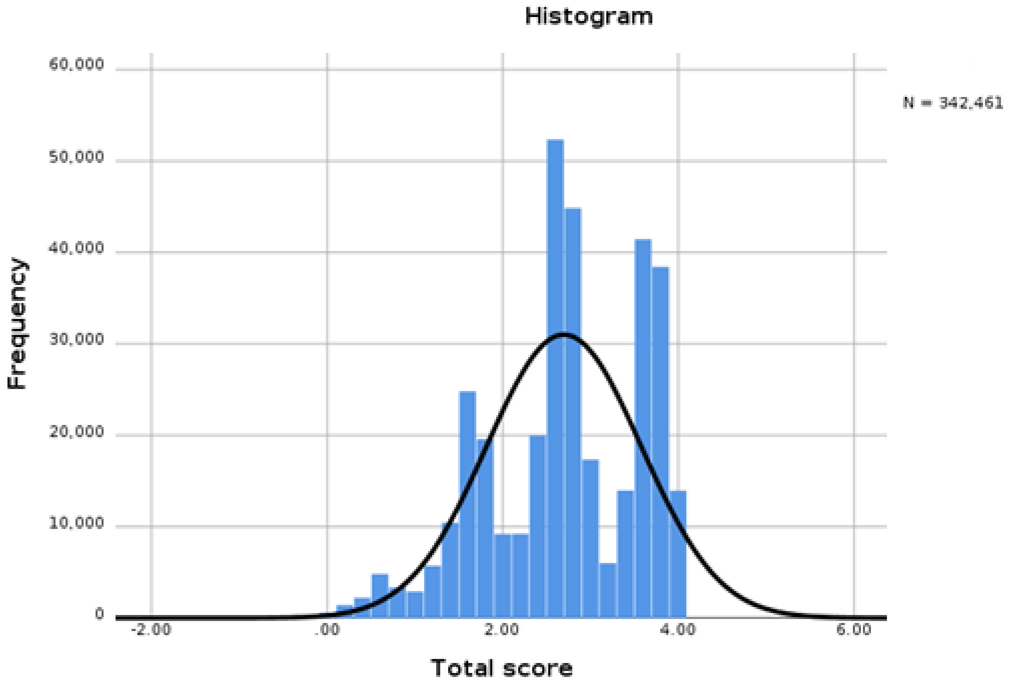

REFERENCES

1 Bycroft C, Freeman C, Petkova D, Band G, Elliott LT, Sharp K, et al. The UK Biobank resource with deep phenotyping and genomic data. Nature. 2018;562(7726):203-09.

2 Sudlow C, Gallacher J, Allen N, Beral V, Burton P, Danesh J, et al. UK biobank: an open access resource for identifying the causes of a wide range of complex diseases of middle and old age. Plos Med. 2015;12(3):e1001779.

3 Howard DM, Adams MJ, Shirali M, Clarke TK, Marioni RE, Davies G, et al. Genome-wide association study of depression phenotypes in UK Biobank identifies variants in excitatory synaptic pathways. Nat Commun. 2018;9(1):1470.

4 Jansen IE, Savage JE, Watanabe K, Bryois J, Williams DM, Steinberg S, et al. Genome-wide meta-analysis identifies new loci and functional pathways influencing Alzheimer's disease risk. Nat Genet. 2019;51(3):404-13.

5 Howard D, Clarke T, Adams M, Hafferty J, Wigmore E, Zeng YN, et al. The Identification of Heterogeneous Genetic Subgroups for Major Depressive Disorder. Eur Neuropsychopharm. 2019;29:S846-S46.

6 Bulik-Sullivan B, Finucane HK, Anttila V, Gusev A, Day FR, Loh PR, et al. An atlas of genetic correlations across human diseases and traits. Nat Genet. 2015;47(11):1236-41.

7 Bulik-Sullivan BK, Loh PR, Finucane HK, Ripke S, Yang J, Schizophrenia Working Group of the Psychiatric Genomics C, et al. LD Score regression distinguishes confounding from polygenicity in genome-wide association studies. Nat Genet. 2015;47(3):291-5.

8 de Leeuw CA, Mooij JM, Heskes T, Posthuma D. MAGMA: generalized gene-set analysis of GWAS data. PLoS Comput Biol. 2015;11(4):e1004219.

9 Grove J, Ripke S, Als TD, Mattheisen M, Walters RK, Won H, et al. Identification of common genetic risk variants for autism spectrum disorder. Nat Genet. 2019;51(3):431-44.

10 Lambert JC, Ibrahim-Verbaas CA, Harold D, Naj AC, Sims R, Bellenguez C, et al. Meta-analysis of 74,046 individuals identifies 11 new susceptibility loci for Alzheimer's disease. Nat Genet. 2013;45(12):1452-8.

11 Ripke S, Neale BM, Corvin A, Walters JTR, Farh KH, Holmans PA, et al. Biological insights from 108 schizophrenia-associated genetic loci. Nature. 2014;511(7510):421-+.

12 Stahl EA, Breen G, Forstner AJ, McQuillin A, Ripke S, Trubetskoy V, et al. Genome-wide association study identifies 30 loci associated with bipolar disorder. Nat Genet. 2019;51(5):793-803.
13 Wray NR, Ripke S, Mattheisen M, Trzaskowski M, Byrne EM, Abdellaoui A, et al. Genome-wide association analyses identify 44 risk variants and refine the genetic architecture of major depression. Nat Genet. 2018;50(5):668-81.
14 Gao JJ, Davis LK, Hart AB, Sanchez-Roige S, Han LD, Cacioppo JT, et al. Genome-Wide Association Study of Loneliness Demonstrates a Role for Common Variation. Neuropsychopharmacol. 2017;42(4):811-21.
15 Stein MB, Chen CY, Jain S, Jensen KP, He F, Heeringa SG, et al. Genetic risk variants for social anxiety. Am J Med Genet B Neuropsychiatr Genet. 2017;174(4):470-82.
16 Finucane HK, Bulik-Sullivan B, Gusev A, Trynka G, Reshef Y, Loh PR, et al. Partitioning heritability by functional annotation using genome-wide association summary statistics. Nat Genet. 2015;47(11):1228-35.
17 Watanabe K, Taskesen E, van Bochoven A, Posthuma D. Functional mapping and annotation of genetic associations with FUMA. Nat Commun. 2017;8(1):1826.
18 Wang K, Li M, Hakonarson H. ANNOVAR: functional annotation of genetic variants from high-throughput sequencing data. Nucleic Acids Res. 2010;38(16):e164.
19 Kircher M, Witten DM, Jain P, O'Roak BJ, Cooper GM, Shendure J. A general framework for estimating the relative pathogenicity of human genetic variants. Nat Genet. 2014;46(3):310-5.
20 Amendola LM, Dorschner MO, Robertson PD, Salama JS, Hart R, Shirts BH, et al. Actionable exomic incidental findings in 6503 participants: challenges of variant classification. Genome Res. 2015;25(3):305-15.
21 Boyle AP, Hong EL, Hariharan M, Cheng Y, Schaub MA, Kasowski M, et al. Annotation of functional variation in personal genomes using RegulomeDB. Genome Res. 2012;22(9):1790-7.
22 Roadmap Epigenomics C, Kundaje A, Meuleman W, Ernst J, Bilenky M, Yen A, et al. Integrative analysis of 111 reference human epigenomes. Nature. 2015;518(7539):317-30.
23 Psych EC, Akbarian S, Liu C, Knowles JA, Vaccarino FM, Farnham PJ, et al. The PsychENCODE project. Nat Neurosci. 2015;18(12):1707-12.
24 Hauberg ME, Zhang W, Giambartolomei C, Franzen O, Morris DL, Vyse TJ, et al. Large-Scale Identification of Common Trait and Disease Variants Affecting Gene Expression. Am J Hum Genet. 2017;101(1):157.
25 Ramasamy A, Trabzuni D, Guelfi S, Varghese V, Smith C, Walker R, et al. Genetic variability in the regulation of gene expression in ten regions of the human brain. Nat Neurosci. 2014;17(10):1418-28.
26 e GP. Enhancing GTEx by bridging the gaps between genotype, gene expression, and disease. Nat Genet. 2017;49(12):1664-70.
27 Giusti-Rodríguez P, Lu L, Yang Y, Crowley CA, Liu X, Ivan Juric, et al. Using three-dimensional regulatory chromatin interactions from adult and fetal cortex to interpret genetic results for psychiatric disorders and cognitive traits. BioRxiv. 2019.
28 Schmitt AD, Hu M, Jung I, Xu Z, Qiu Y, Tan CL, et al. A Compendium of Chromatin Contact Maps Reveals Spatially Active Regions in the Human Genome. Cell Rep. 2016;17(8):2042-59.
29 Choi SW, O'Reilly PF. PRSice-2: Polygenic Risk Score software for biobank-scale data. Gigascience. 2019;8(7).
30 Penninx BW, Beekman AT, Smit JH, Zitman FG, Nolen WA, Spinhoven P, et al. The Netherlands Study of Depression and Anxiety (NESDA): rationale, objectives and methods. Int J Methods Psychiatr Res. 2008;17(3):121-40.
31 Fabbri C, Kasper S, Kautzky A, Bartova L, Dold M, Zohar J, et al. Genome-wide association study of treatment-resistance in depression and meta-analysis of three independent samples. Br J Psychiatry. 2019;214(1):36-41.

32 Garriock HA, Kraft JB, Shyn SI, Peters EJ, Yokoyama JS, Jenkins GD, et al. A genomewide association study of citalopram response in major depressive disorder. Biol Psychiatry. 2010;67(2):133-8.
33 Sullivan PF, Lin D, Tzeng JY, van den Oord E, Perkins D, Stroup TS, et al. Genomewide association for schizophrenia in the CATIE study: results of stage 1. Mol Psychiatry. 2008;13(6):570-84.
34 Sklar P, Smoller JW, Fan J, Ferreira MA, Perlis RH, Chambert K, et al. Whole-genome association study of bipolar disorder. Mol Psychiatry. 2008;13(6):558-69.
35 de Beurs E, den Hollander-Gijsman ME, van Rood YR, van der Wee NJ, Giltay EJ, van Noorden MS, et al. Routine outcome monitoring in the Netherlands: practical experiences with a web-based strategy for the assessment of treatment outcome in clinical practice. Clin Psychol Psychother. 2011;18(1):1-12.
36 Vreeburg SA, Zitman FG, van Pelt J, Derijk RH, Verhagen JC, van Dyck R, et al. Salivary cortisol levels in persons with and without different anxiety disorders. Psychosom Med. 2010;72(4):340-7.
37 van Kampen D, de Beurs E, Andrea H. A short form of the Dimensional Assessment of Personality Pathology-Basic Questionnaire (DAPP-BQ): the DAPP-SF. Psychiatry Res. 2008;160(1):115-28.
38 Derogatis LR, Melisaratos N. The Brief Symptom Inventory: an introductory report. Psychol Med. 1983;13(3):595-605.
39 Ware JE, Jr., Sherbourne CD. The MOS 36-item short-form health survey (SF-36). I. Conceptual framework and item selection. Med Care. 1992;30(6):473-83.

SUPPLEMENTARY FIGURES

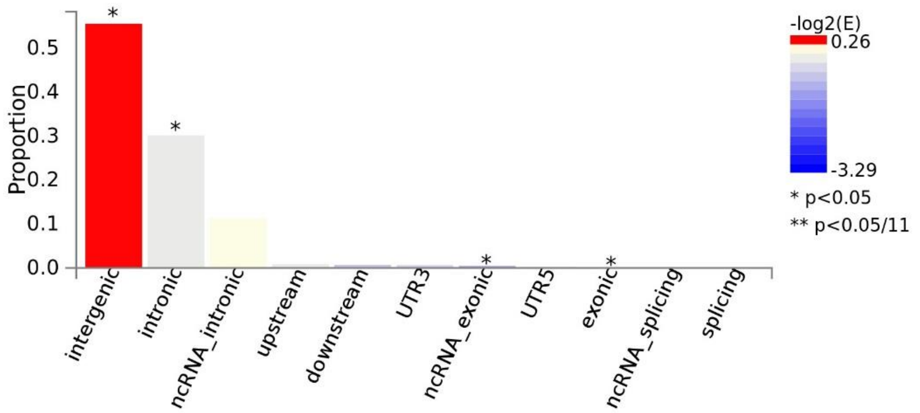

Supplementary Figure S1. Positional annotation of candidate SNPs. Shown in the histogram is the proportion of SNPs per location. When SNPs have more than one (different) annotations, they are counted for each annotation.

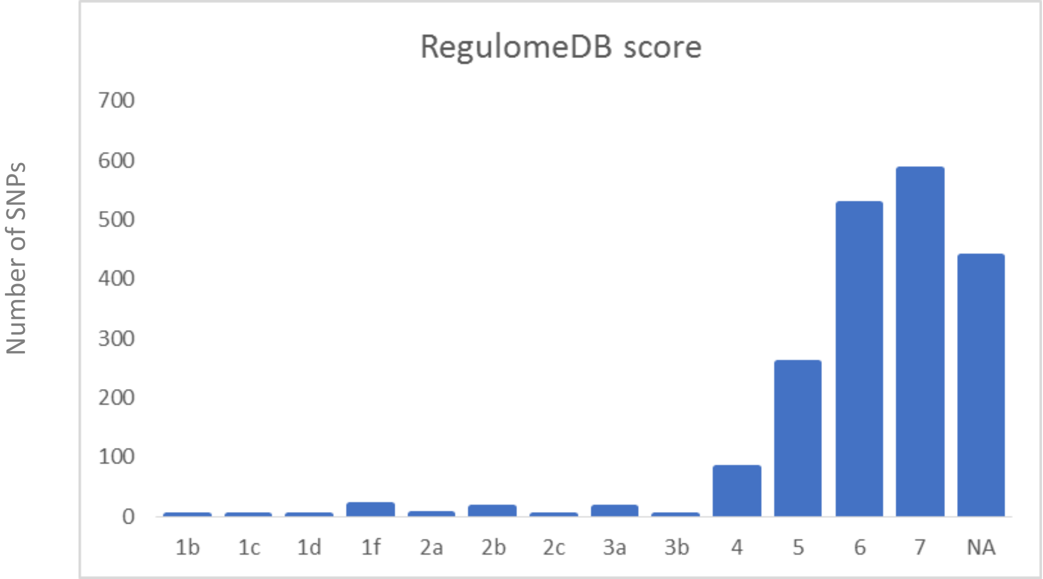

Supplementary Figure S2. RegulomeDB scores. RegulomeDB scores represent predicted regulatory functionality of SNPs based on eQTLs and chromatin marks, ranging from 1a to 7, with lower numbers indicating higher likelihood of having a regulatory function. Regulome scores are assigned according to the following pattern: 1a = eQTL + transcription factor binding + matched transcription factor motif + matched DNase footprint + DNase peak (not present in our data); 1b = eQTL + transcription factor binding + any motif + DNase footprint + DNase peak; 1c = eQTL + transcription factor binding + matched transcription factor motif + DNase peak; 1d = eQTL + transcription factor binding + any motif + DNase

peak; 1e = eQTL + transcription factor binding + matched transcription factor motif; 1f = eQTL + transcription factor binding/DNase peak; 2a = transcription factor binding + matched transcription factor motif + matched DNase footprint + DNase peak; 2b = transcription factor binding + any motif + DNase footprint + DNase peak; 2c = transcription factor binding + matched transcription factor motif + DNase peak; 3a = transcription factor binding + any motif + DNase peak; 3b = transcription factor binding + matched transcription factor motif; 4 = transcription factor binding + DNase peak; 5 = transcription factor binding or DNase peak; 6 = other; 7 = no information available, NA = variables not present in the database.

Chromosome 2

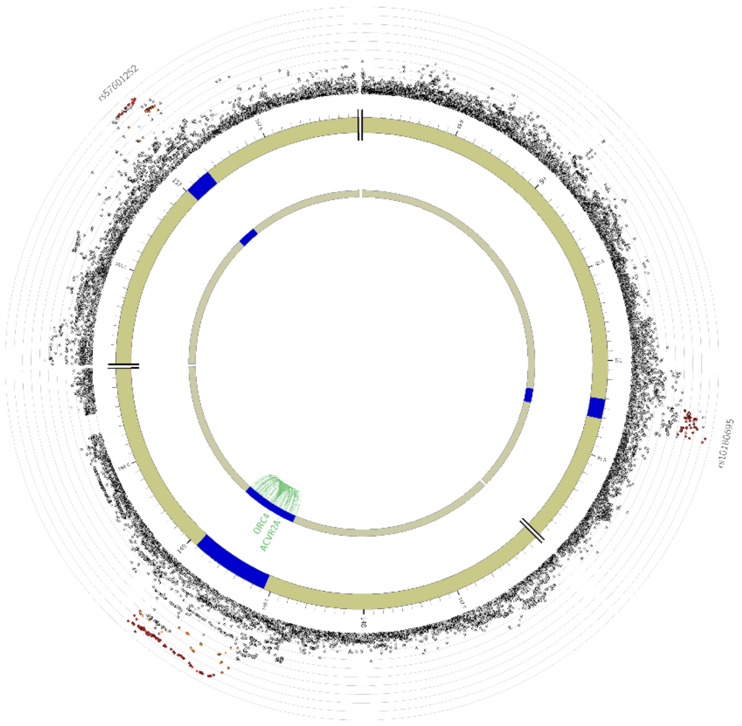

Chromosome 3

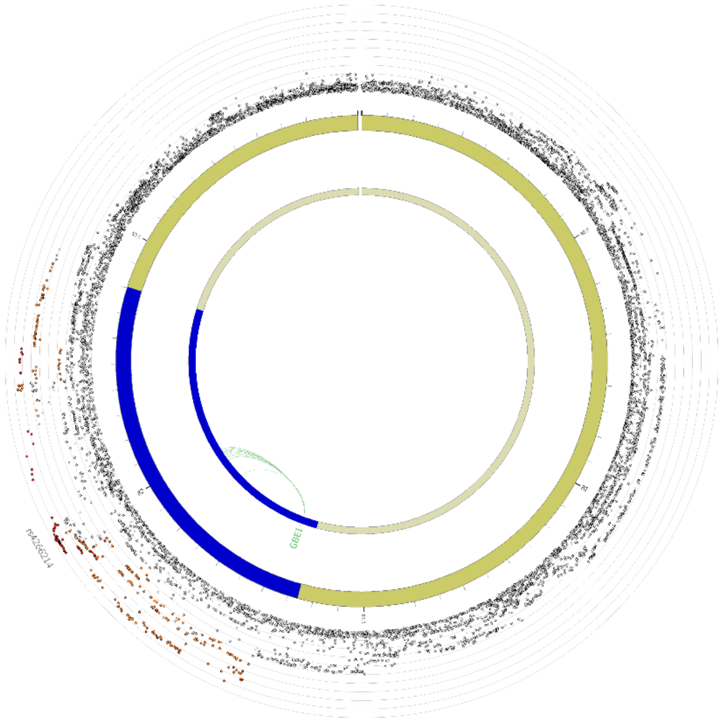

Chromosome 5

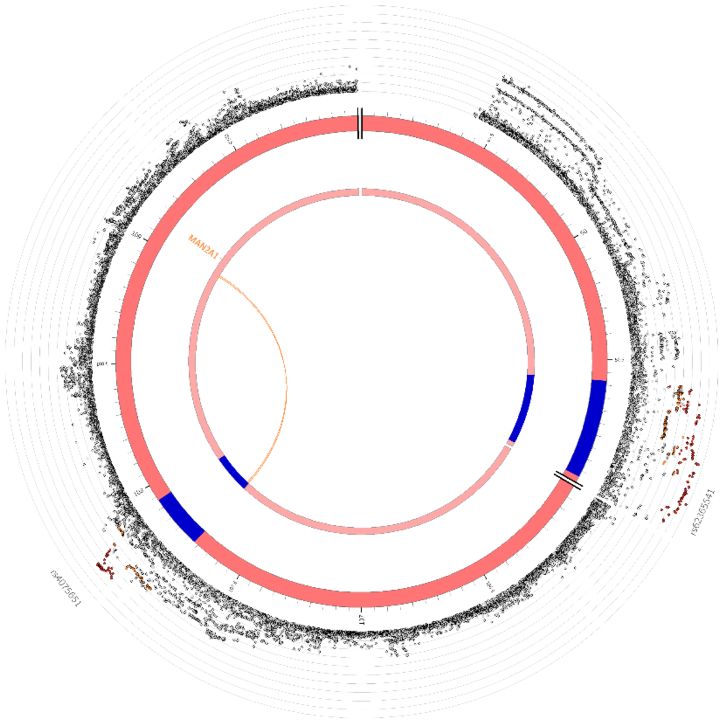

Chromosome 6

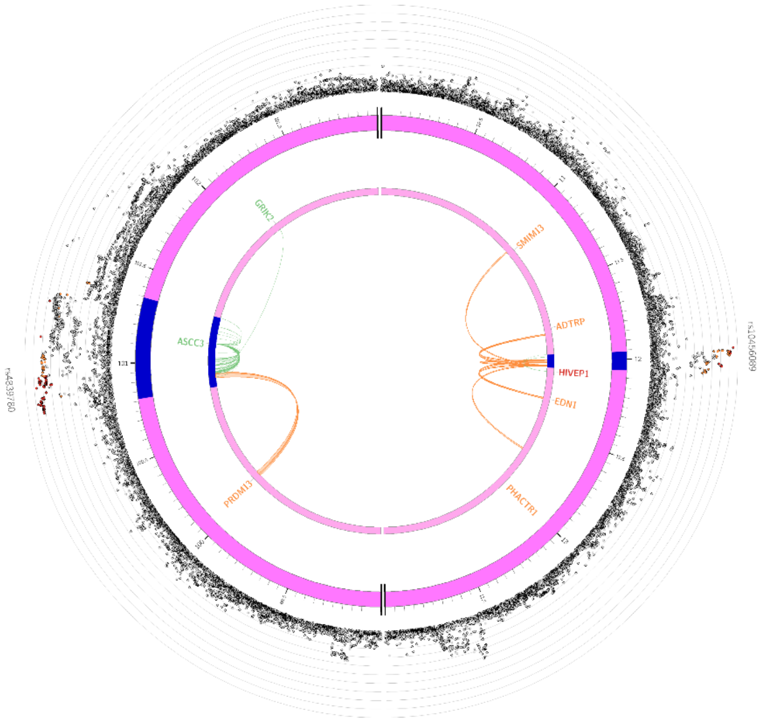

Chromosome 7

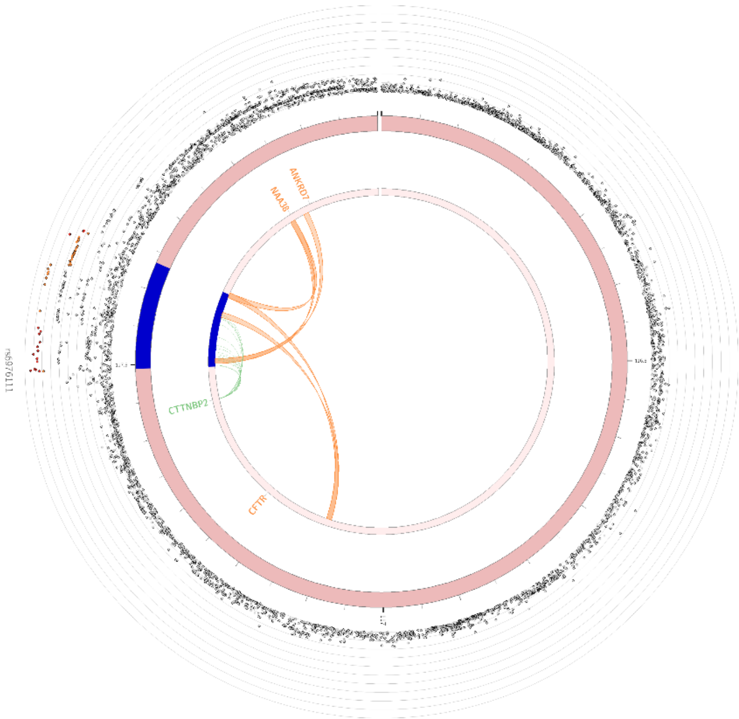

Chromosome 9

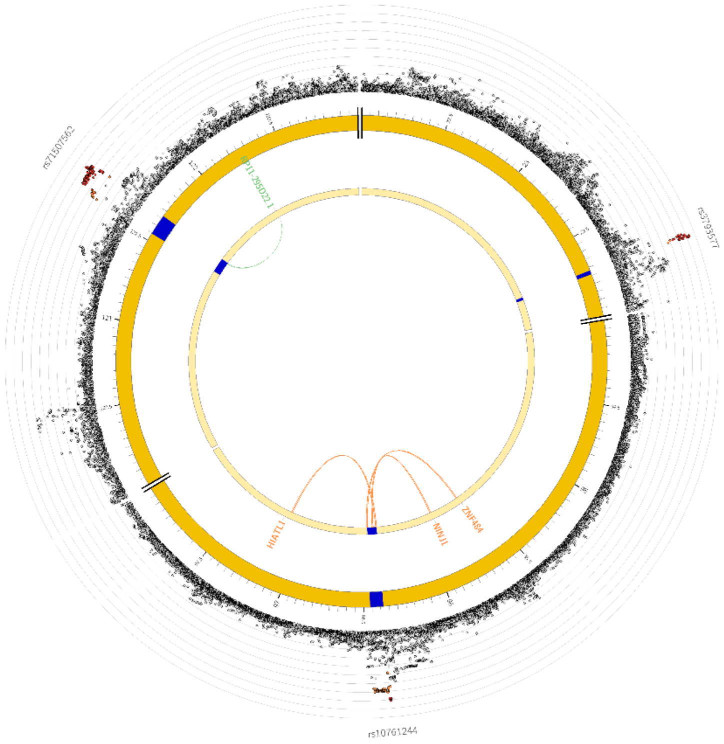

Chromosome 11

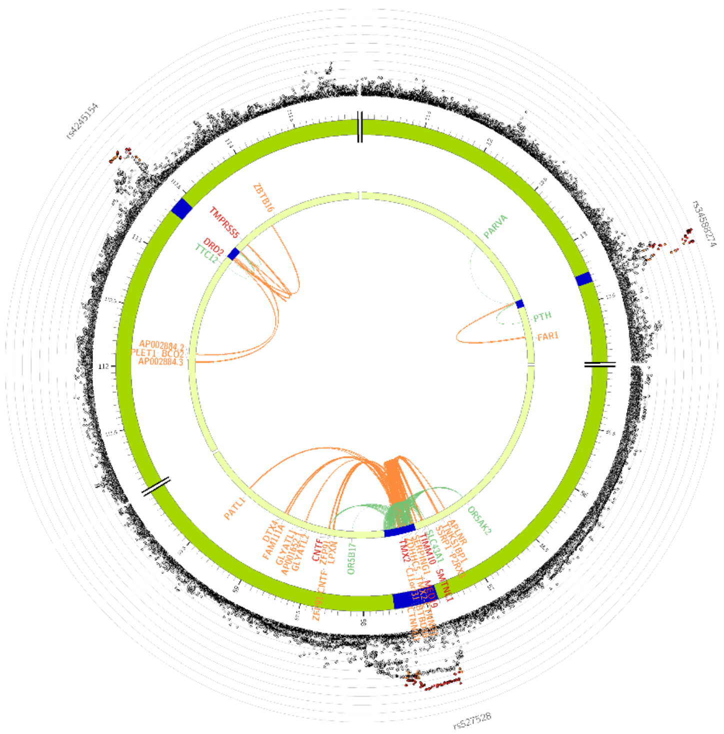

Chromosome 12

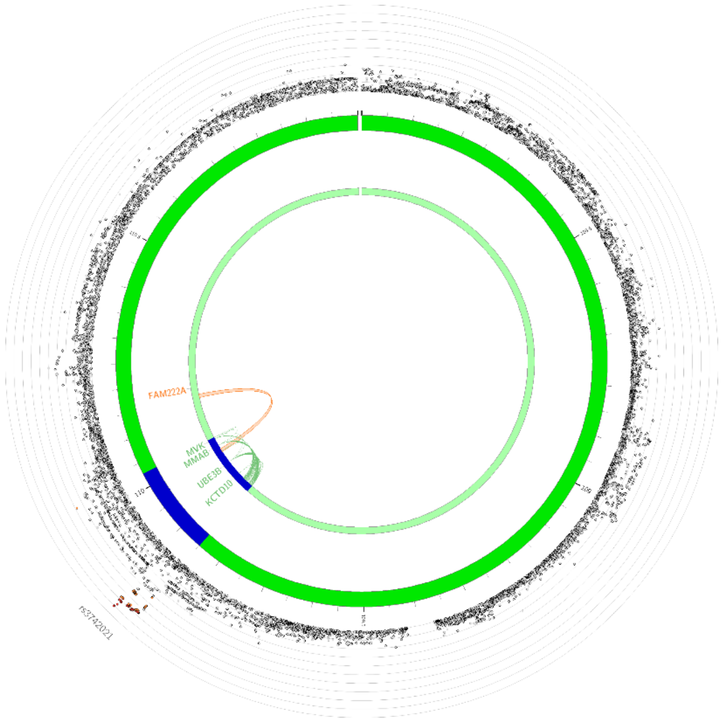

Chromosome 14

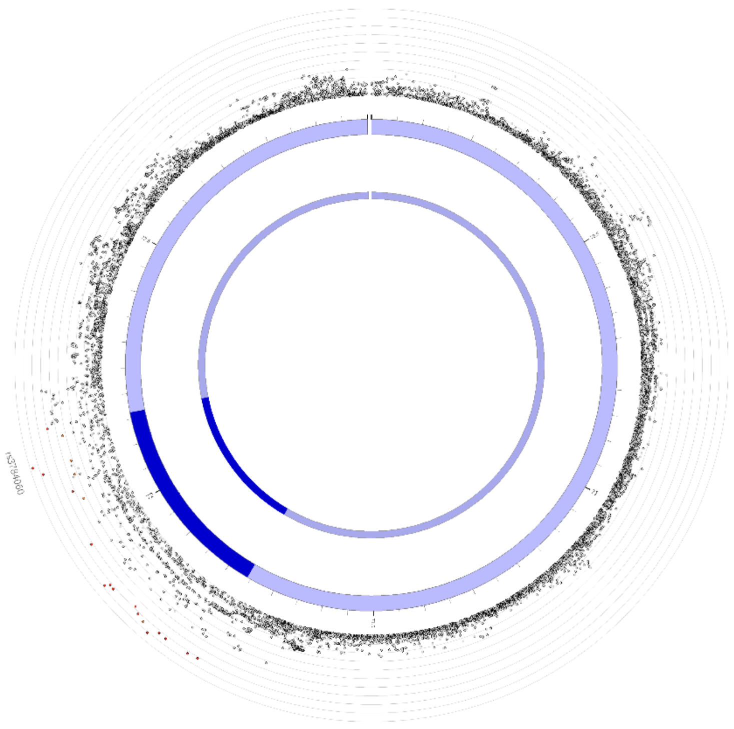

Chromosome 18

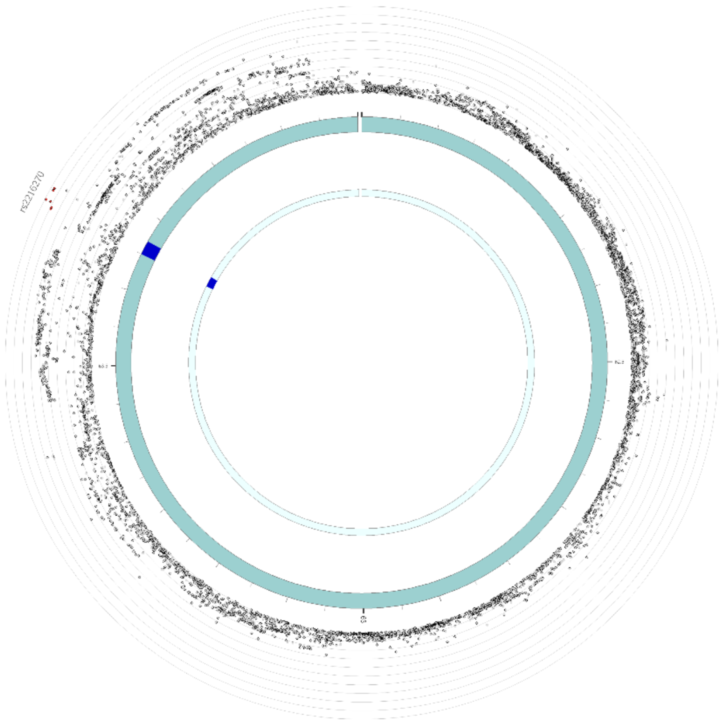

Supplementary Figure S3 Circos plots of chromatin interactions and eQTLs**.** The most outer layer shows the Manhattan plot. Only SNPs with P < 0.05 are displayed. SNPs in genomic risk loci are color-coded as a function of their maximum r^2 to one of the independent significant SNPs in the locus, as follows: red ($r^2 > 0.8$), orange ($r^2 > 0.6$), green ($r^2 > 0.4$) and blue ($r^2 > 0.2$). SNPs that are not in LD with any of the independent significant SNPs (with $r^2 \leq 0.2$) are grey. The rsID of the top-SNPs in each risk locus are displayed in the most outer layer. Y-axis indicates the -log10(P-value) of the SNPs. The second layer contains the chromosome, represented as a ring. Genomic risk loci are highlighted in blue. Only genes mapped by either chromatin interaction and/or eQTLs are displayed. If the gene is mapped only by chromatin interactions or only by eQTLs, the ring is colored orange or green, respectively. When the gene is mapped by both, it is colored red. The third layer contains another chromosome ring. This is the same as the second layer but without coordinates to make it easy to align position of genes with genomic coordinate. Links colored orange represent chromatin interactions. Links colored green represent eQTLs. See Github repository FUMA circos plot for more details.

A.

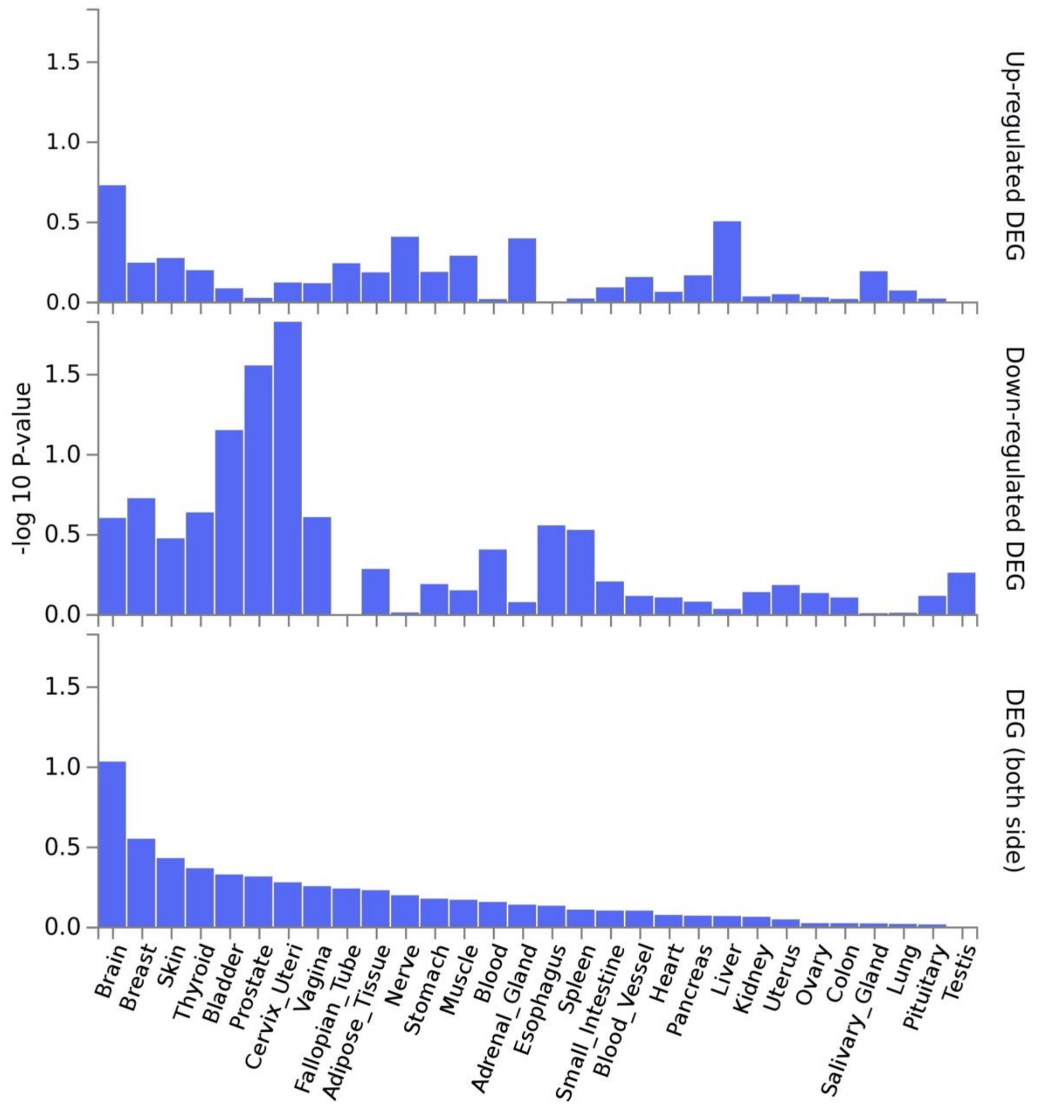
Up-regulated DEG
Down-regulated DEG
DEG (both side)
-log 10 P-value
1.5
1.0
0.5
0.0
Brain
Breast
Skin
Thyroid
Bladder
Prostate
Cervix_Uteri
Vagina
Fallopian_Tube
Adipose_Tissue
Nerve
Stomach
Muscle
Blood
Adrenal_Gland
Esophagus
Spleen
Small_Intestine
Blood_Vessel
Heart
Pancreas
Liver
Kidney
Uterus
Ovary
Colon
Salivary_Gland
Lung
Pituitary
Testis

B.

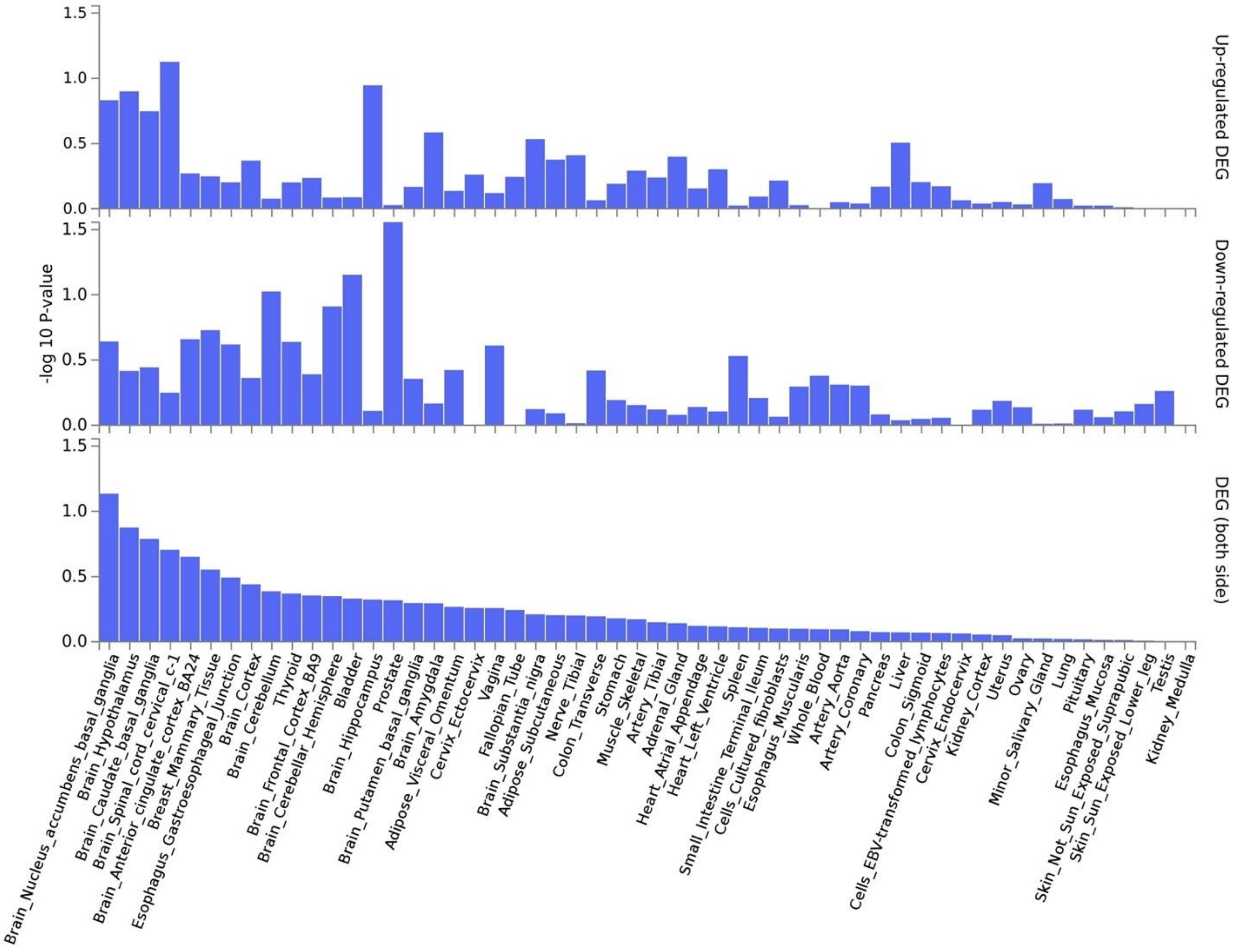
-log 10 P-value
Up-regulated DEG
Down-regulated DEG
DEG (both side)
0.0
0.5
1.0
1.5
Brain_Nucleus_accumbens_basal_ganglia
Brain_Hypothalamus
Brain_Caudate_basal_ganglia
Brain_Spinal_cord_cervical_c-1
Brain_Anterior_cingulate_cortex_BA24
Breast_Mammary_Tissue
Esophagus_Gastroesophageal_Junction
Brain_Cortex
Brain_Cerebellum
Thyroid
Brain_Frontal_Cortex_BA9
Brain_Cerebellar_Hemisphere
Bladder
Brain_Hippocampus
Prostate
Brain_Putamen_basal_ganglia
Brain_Amygdala
Adipose_Visceral_Omentum
Cervix_Ectocervix
Vagina
Fallopian_Tube
Brain_Substantia_nigra
Adipose_Subcutaneous
Nerve_Tibial
Colon_Transverse
Stomach
Muscle_Skeletal
Artery_Tibial
Adrenal_Gland
Heart_Atrial_Appendage
Heart_Left_Ventricle
Spleen
Small_Intestine_Terminal_Ileum
Cells_Cultured_fibroblasts
Esophagus_Muscularis
Whole_Blood
Artery_Aorta
Artery_Coronary
Pancreas
Liver
Colon_Sigmoid
Cells_EBV-transformed_lymphocytes
Cervix_Endocervix
Kidney_Cortex
Uterus
Ovary
Minor_Salivary_Gland
Lung
Pituitary
Esophagus_Mucosa
Skin_Not_Sun_Exposed_Suprapubic
Skin_Sun_Exposed_Lower_leg
Testis
Kidney_Medulla

c.

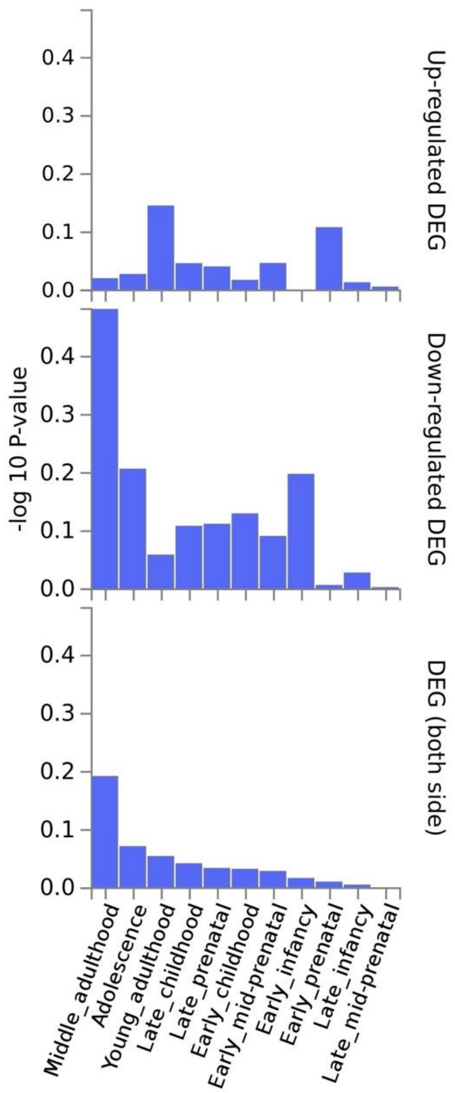
Up-regulated DEG
Down-regulated DEG
DEG (both side)
-log 10 P-value
0.0
0.1
0.2
0.3
0.4
Middle_adulthood
Adolescence
Young_adulthood
Late_childhood
Late_prenatal
Early_childhood
Early_mid-prenatal
Early_infancy
Early_prenatal
Late_infancy
Late_mid-prenatal

D.

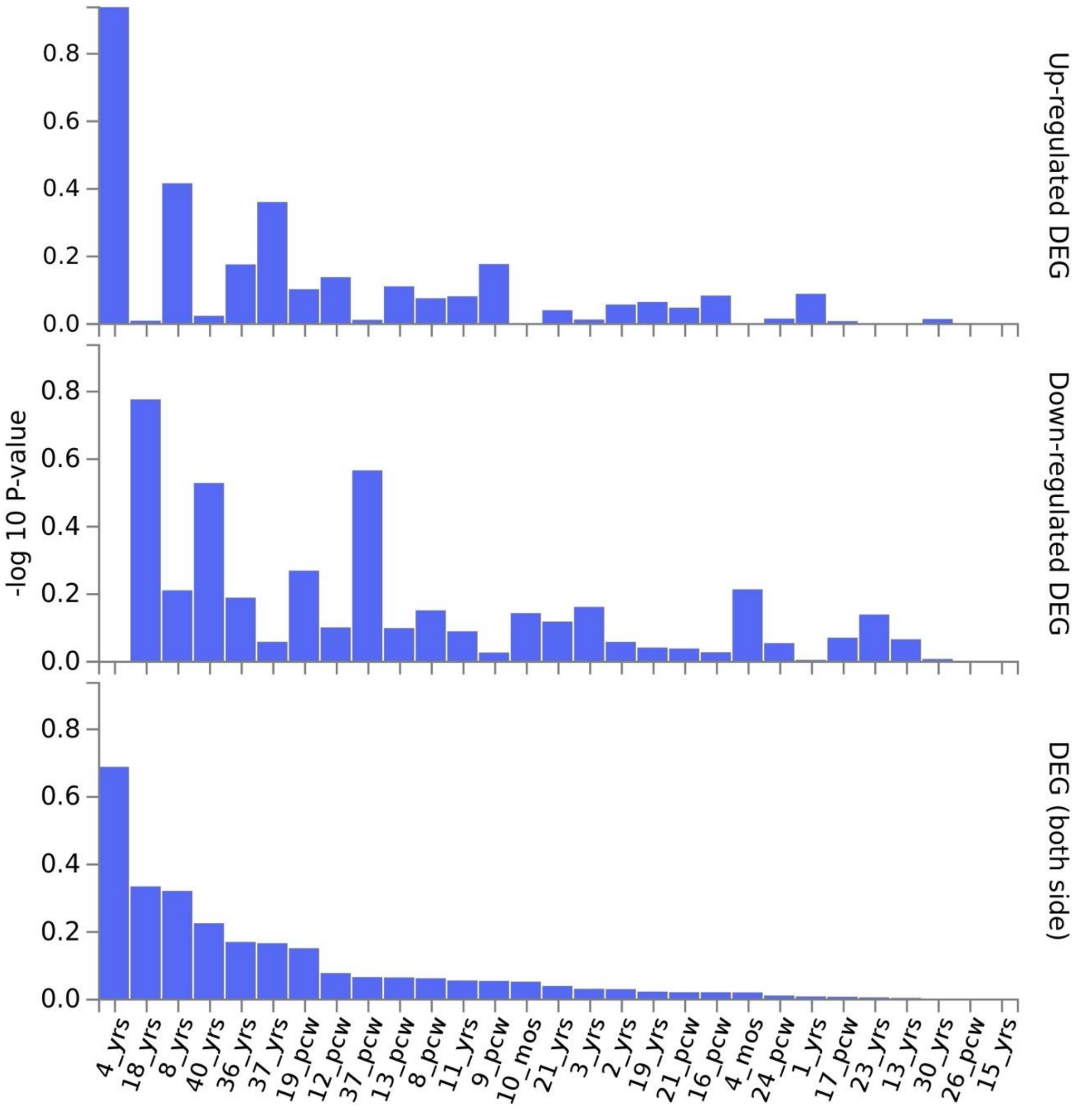

Supplementary Figure S4 Tissue expression enrichment analyses based on the 76 FUMA-mapped genes potentially implicated in sociability. Enrichment analyses of differentially expressed genes (DEG) were performed in FUMA as part of the GENE2FUNC procedure, using default parameters, across **(A)** 30 general tissue types from the GTEx database v8, **(B)** 54 specific tissue types from the GTEx database v8, **(C)** 11 general developmental stages of brain samples from the BrainSpan data and **(D)** 29 different ages of brain samples from the BrainSpan data. For each set of analyses, results are also show for up-regulated DEGs and down-regulated DEGs, in addition to the DEG (for both sides), by taking the sign of t-statistics into account. Input genes were tested against each of the DEG sets using the hypergeometric test. No significant enriched DEG sets (P<0.05, Bonferroni-corrected) were observed.

A.

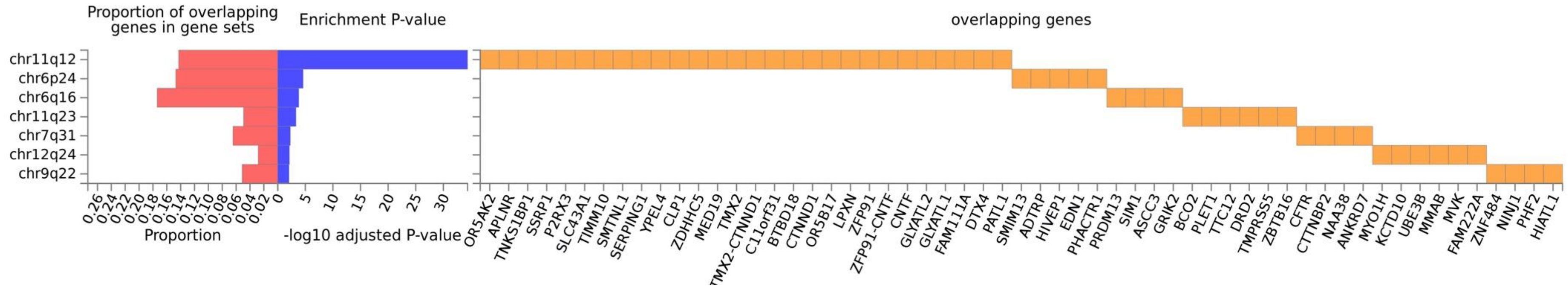
Proportion of overlapping genes in gene sets
Enrichment P-value
overlapping genes
chr11q12
chr6p24
chr6q16
chr11q23
chr7q31
chr12q24
chr9q22
0.26 0.24 0.22 0.20 0.18 0.16 0.14 0.12 0.10 0.08 0.06 0.04 0.02 0
Proportion
0 5 10 15 20 25 30
-log10 adjusted P-value
OR5AK2
APLNR
TNKS1BP1
SSRP1
P2RX3
SLC43A1
TIMM10
SMTNL1
SERPING1
YPEL4
CLP1
ZDHHC5
MED19
TMX2
TMX2-CTNND1
C11orf31
BTBD18
CTNND1
OR5B17
LPXN
ZFP91
ZFP91-CNTF
CNTF
GLYATL2
GLYATL1
FAM111A
DTX4
PATL1
SMIM13
ADTRP
HIVEP1
EDN1
PHACTR1
PRDM13
SIM1
ASCC3
GRIK2
BCO2
PLET1
TTC12
DRD2
TMPRSS5
ZBTB16
CFTR
CTTNBP2
NAA38
ANKRD7
MYO1H
KCTD10
UBE3B
MMAB
MVK
FAM222A
ZNF484
NINJ1
PHF2
HIATL1

B.

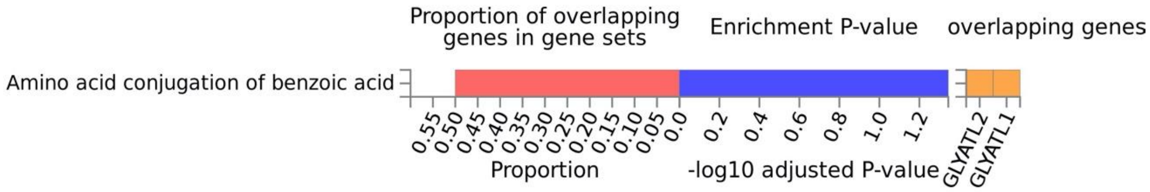
Proportion of overlapping genes in gene sets
Enrichment P-value
overlapping genes
Amino acid conjugation of benzoic acid
0.55 0.50 0.45 0.40 0.35 0.30 0.25 0.20 0.15 0.10 0.05 0.0
Proportion
0.2 0.4 0.6 0.8 1.0 1.2
-log10 adjusted P-value
GLYATL2
GLYATL1

C.

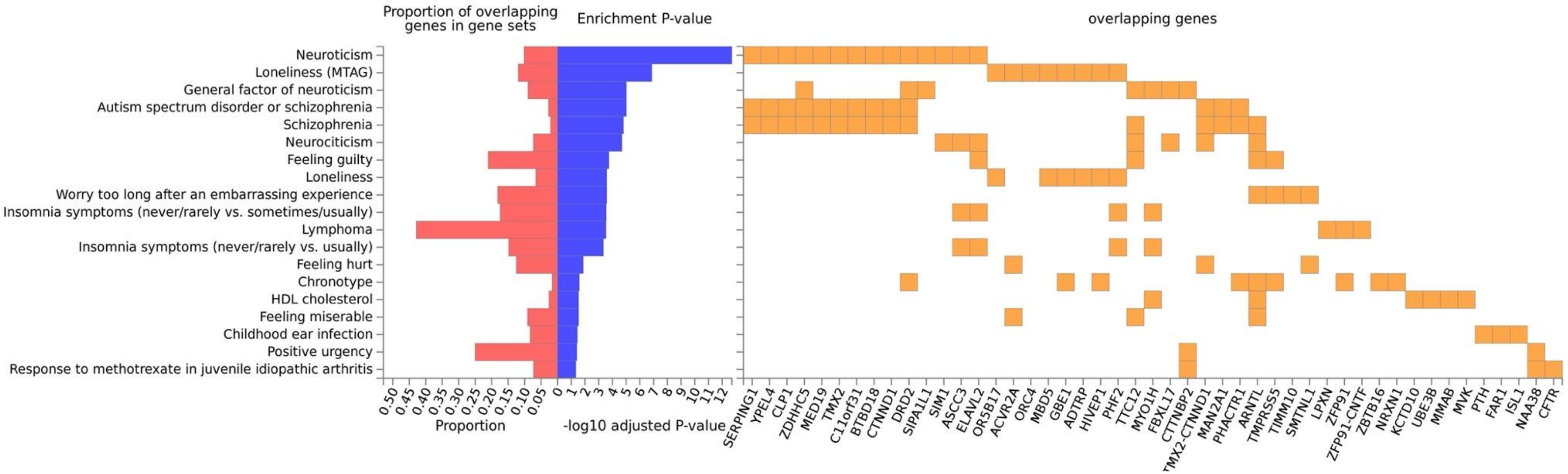
Proportion of overlapping genes in gene sets
Enrichment P-value
overlapping genes
Neuroticism
Loneliness (MTAG)
General factor of neuroticism
Autism spectrum disorder or schizophrenia
Schizophrenia
Neurociticism
Feeling guilty
Loneliness
Worry too long after an embarrassing experience
Insomnia symptoms (never/rarely vs. sometimes/usually)
Lymphoma
Insomnia symptoms (never/rarely vs. usually)
Feeling hurt
Chronotype
HDL cholesterol
Feeling miserable
Childhood ear infection
Positive urgency
Response to methotrexate in juvenile idiopathic arthritis
0.50 0.45 0.40 0.35 0.30 0.25 0.20 0.15 0.10 0.05 0
Proportion
1 2 3 4 5 6 7 8 9 10 11 12
-log10 adjusted P-value
SERPING1
YPEL4
CLP1
ZDHHC5
MED19
TMX2
C11orf31
BTBD18
CTNND1
DRD2
SIPA1L1
SIM1
ASCC3
ELAVL2
OR5B17
ACVR2A
ORC4
MBD5
GBE1
ADTRP
HIVEP1
PHF2
TTC12
MYO1H
FBXL17
CTTNBP2
TMX2-CTNND1
MAN2A1
PHACTR1
ARNTL
TMPRSS5
TIMM10
SMTNL1
LPXN
ZFP91
ZFP91-CNTF
ZBTB16
NRXN1
KCTD10
UBE3B
MMAB
MVK
PTH
FAR1
ISL1
NAA38
CFTR

Supplementary Figure S5 Significantly enriched gene-sets identified using as query the 76 FUMA-mapped genes potentially implicated in sociability. Hypergeometric tests were performed in FUMA using default parameters: adjusted P-value (FDR) was obtained by the Benjamini–Hochberg method; significance threshold of adjusted P-value was 0.05; the minimum number of overlapping genes between the input and the tested gene sets is set on two genes. Results are grouped according to the source of the pre-defined gene set. **A.** Positional gene sets (MsigDB c1). **B.** WikiPathway gene set. **C.** GWAS catalog reported genes.

SUPPLEMENTARY TABLES

Supplementary Table 1. PRISM cohorts included in the case-only PRS analyses.

Cohort	n	Disorder	Behavioural phenotype
NESDA	1705	Lifetime diagnosis of major depressive disorder and/or anxiety	De Jong Gierveld loneliness scale
GRSD	1116	Major Depressive Disorder	Social and family functioning items of the Sheenen scale
STARD	1146	Major Depressive Disorder	Social activities and relationships from the Work and Social Adjustment Scale (WSAS)
CATIE	478	Schizophrenia	Passive/apathetic social withdrawal and active social avoidance of the Positive and Negative Syndrome Scale (PANSS)
STEP-BD	701	Bipolar Disorder	Interpersonal relations with relatives and interpersonal relations with friends from the Range of Impaired Functioning Tool (LRIFT)
ROM study	427	One or more Mood, Anxiety or Somatoform disorders	The Dimensional Assessment of Personality Pathology-Short Form (DAPP-SF) Social avoidance subscale, Brief Symptom Inventory (BSI) and the Short Form-36 Social functioning subscale

Supplementary Table 2. Outcome phenotypic analyses.

	Mean	Standard error	Confidence Interval	P-value
Not affected	2.729	0.002	2.726-2.732	-
ASD	1.981	0.135	1.717-2.246	4.12E-4
AD-by-proxy	2.701	0.004	2.693-2.710	1.05E-8
BPD	2.326	0.032	2.263-2.389	4.65E-28
MDD	2.220	0.009	2.202-2.238	<1E-28
SCZ	2.151	0.044	2.065-2.237	1.56E-27

Note: Mean, standard error, confidence interval and p-value for the phenotypic analyses comparing the not affected group to individuals with autism spectrum disorder (ASD), Alzheimer's Disease (AD) -by-proxy, BiPolar Disorder (BPD), Major Depressive Disorder (MDD) and SCiZophrenia (SCZ).

Supplementary Table 3. Lead SNPs with p-values from the split-half validation and not affected analyses.

Lead SNPs	1.0	1.1	2.0	2.1	3.0	3.1	4.0	4.1	5.0	5.1	Not Affected
rs10180695	0.000222886	3.81043e-05	8.82303e-05	8.30185e-05	4.78534e-05	0.000170333	4.93894e-05	0.000175004	4.7886e-06	0.00108509	5.28666e-06
rs202220108	3.23239e-07	0.00630981	3.23192e-06	0.00166986	3.45484e-05	0.00021632	0.00350474	1.02686e-06	1.74165e-06	0.00224407	2.11264e-06
rs57601252	6.77958e-07	0.00486756	0.000579747	1.33684e-05	0.00102539	7.46895e-06	0.00296319	9.9482e-07	0.00379655	1.51727e-06	1.34056e-07
rs4266214	0.00139117	9.04529e-08	0.00234441	3.39663e-08	3.41974e-07	0.000543444	3.99936e-05	8.64915e-06	6.55897e-05	6.54688e-06	5.52953e-06
rs62365541	4.00211e-08	6.01995e-05	9.53032e-08	2.38682e-05	4.59101e-05	6.47652e-08	3.34331e-07	9.08024e-06	3.23314e-06	1.62666e-06	1.99929e-10
rs4075651	4.26383e-07	0.00190128	9.60724e-05	2.44448e-05	1.12423e-07	0.00527932	0.00374923	1.89718e-07	2.01234e-06	0.000753375	3.58224e-08

rs10456089	0.00030673	1.23212e-06	5.86212e-05	1.44502e-05	4.13962e-05	1.28888e-05	6.4469e-05	1.02336e-05	1.60797e-06	0.000302213	4.14047e-07
rs4839780	7.68668e-09	0.0243191	1.78221e-05	0.000162533	4.96045e-06	0.000480647	3.39981e-05	9.35793e-05	1.61467e-06	0.00108487	1.90015e-07
rs34979551	0.00250786	4.76328e-07	4.60867e-05	8.48154e-05	8.34066e-05	4.23544e-05	0.000950076	1.80208e-06	6.06646e-07	0.00226847	2.11222e-07
rs6976111	9.50484e-07	9.87955e-05	0.000972854	3.207e-08	1.58275e-05	6.96567e-06	9.96051e-05	8.58103e-07	8.1314e-06	1.18004e-05	5.88257e-07
rs3793577	0.000167511	4.17932e-07	2.69818e-05	4.19719e-06	3.03465e-05	3.04103e-06	1.90543e-06	4.63736e-05	0.000130515	6.57406e-07	3.27556e-08
rs10761244	0.00120077	8.02226e-07	0.000112807	1.99484e-05	0.0020446	4.21534e-07	8.97422e-07	0.0010392	3.47806e-05	6.29227e-05	9.81038e-08
rs71507562	2.15726e-06	0.00140564	0.00177737	1.28612e-06	3.8843e-06	0.000708262	8.98351e-06	0.000438834	1.60183e-05	0.000198062	1.5161e-05
rs34588274	7.49441e-08	3.00921e-07	4.91911e-11	6.67814e-05	2.64506e-09	4.71615e-06	1.84105e-09	7.13064e-06	1.40683e-07	1.64668e-07	9.05112e-10
rs527528	1.57371e-06	5.78132e-05	6.12168e-06	1.39896e-05	4.10069e-07	0.000138345	1.05099e-07	0.000386852	3.90497e-06	1.97712e-05	6.15352e-06
rs4245154	5.02158e-07	0.00142873	0.000223106	5.43597e-06	0.00216752	2.67659e-07	0.00435997	1.16913e-07	0.000557834	2.19748e-06	4.59753e-07
rs3742021	7.56999e-05	2.70369e-05	2.7609e-06	0.000504432	6.76715e-05	2.71325e-05	3.34113e-06	0.000426676	0.000188169	9.02683e-06	5.13813e-08
rs3784060	0.00862335	8.29418e-08	0.000577624	5.71896e-06	0.000308454	1.50502e-05	7.74137e-07	0.00262093	0.000443027	8.7807e-06	1.49264e-07
rs2216270	8.76425e-06	0.000306867	0.00272291	4.01544e-07	1.60518e-05	0.000183667	5.05762e-05	6.30054e-05	0.00159604	9.3324e-07	3.87524e-07

Supplementary Table 4. Lead SNPs with p-values from the single question GWAS.

**Lead SNPs**	**Q1**	**Q2**	**Q3**	**Q4**
rs10180695	0.810337	0.000522994	0.000891911	3.76603e-05
rs202220108	0.451854	0.0605746	0.000113599	4.63714e-07
rs57601252	0.785664	0.0165675	3.15934e-07	0.0030558
rs4266214	0.0000212	0.233621	0.00013918	3.19031e-07
rs62365541	0.0096357	0.000106267	2.59728e-06	0.00129168
rs4075651	0.89016	0.0116384	1.00706e-07	0.000740789
rs10456089	0.032227	0.675416	8.83765e-07	2.16225e-08
rs4839780	0.357883	0.00448775	0.00015498	2.10638e-05
rs34979551	0.452202	3.16707e-05	1.10335e-05	0.0546477
rs6976111	0.0544169	0.000403443	3.43442e-06	0.00291351
rs3793577	0.772727	6.12863e-06	1.70874e-05	0.00155214
rs10761244	0.101212	0.00377435	0.015117	1.09669e-08
rs71507562	0.000558578	0.00074455	0.0368573	2.24977e-05
rs34588274	0.35594	0.179519	1.67344e-17	8.69719e-05
rs527528	0.0225053	0.00130828	3.49997e-11	0.00123939
rs4245154	0.132971	0.0321749	8.92356e-09	0.0157775
rs3742021	0.02488	0.0317345	1.44113e-09	0.115512
rs3784060	2.09E-07	0.0250271	0.00355924	0.000406987
rs2216270	4.83E-06	0.000174289	0.00505455	0.0195014

Supplementary Table 5. Genome-wide significant genes without flanking regions. MAGMA results from the main analysis as well as the results for the unaffected only analyses.

Gene	MAIN	Not Affected
TMX2	8.8257e-10	1,4131E-06
MBD5	1.0812e-06	0,000024374
GRM8	1.8984e-07	0,000033146
C11orf31	1.3489e-08	7,3434E-06
TTC12	1.6515e-06	3,1186E-07
SERPING1	2.1128e-07	0,000017412
BAIAP2	4.402e-07	0,0016159
ASCC3	9.0347e-08	4,3389E-06
STAU1	9.7298e-07	0,00001801
CDH7	1.966e-06	0,00011397
MYO1H	1.7246e-07	1,4444E-06
SOX5	7.5792e-08	7,3494E-07
SIM1	2.8066e-09	1,7474E-08
PPP6C	2.0489e-06	0,00016324
CTNND1	6.7831e-11	7,9894E-08
STK4	2.2325e-06	6,0864E-06
DDX39B	1.7866e-09	4,0209E-06
FNBP4	2.2727e-06	0,000040278
CSE1L	5.5622e-08	7,5637E-07
OR9Q1	2.304e-07	8,5756E-06
TLK2	1.4001e-07	6,9446E-07
TAOK3	9.0697e-07	3,6694E-08
MED19	7.1544e-08	2,5564E-06
MCCD1	1.5314e-08	0,000016483
GLIS3	6.982e-08	0,0037867
MRC2	3.841e-07	4,5781E-06
ELAVL2	1.6748e-11	5,3422E-09
PRICKLE1	2.3494e-06	0,0018109
SYBU	1.1816e-06	0,0005218
ASXL3	6.8612e-10	2,2991E-08
CELF4	7.1892e-07	0,000008845
NECAB1	1.2363e-06	0,000019926
FBXL17	3.485e-08	0,000033822
ANKRD10	4.2452e-07	3,0834E-06
KCNJ3	1.6831e-07	3,5251E-06
GBE1	9.3144e-09	0,000015787
RABEPK	3.721e-07	0,000034769
CCDC71	2.2521e-06	0,00013401
PCDH9	4.9682e-07	0,000001317

CCDC36	4.813e-07	0,00002348
HLA-B	7.355e-08	5,4424E-06
URI1	4.6187e-07	0,000001225
ISL1	3.9265e-08	7,6058E-07
ZDHHC5	7.6063e-11	1,2479E-07
SUDS3	9.9904e-08	4,5307E-08
PHF2	7.2294e-09	1,4836E-07
ARPP21	1.7346e-07	7,8668E-08
C8orf88	3.0761e-07	5,2693E-06
DRD2	1.1167e-11	7,2838E-11
TMEM55A	5.8597e-07	0,000015876
SGCZ	8.19e-09	0,000013442
HIST1H2BD	9.7724e-07	0,00075241
ANKK1	1.6303e-06	7,9215E-07
ARNTL	9.9948e-15	4,817E-11
GATAD2A	4.2469e-07	0,0019039
PFDN1	5.5446e-07	0,000010436

Supplementary Table 6. PRS results with sociability GWAS as base and PRISM cohorts measuring social phenotypes within cases with psychiatric disorders as target. Shown in this table are the best SNP P-value thresholds (PT) for the PRSice analyses between the sociability PRS and the social behavior outcomes in the PRISM datasets, the association P-values (P-value), the variance explained in the target sample phenotypes (R2), and the number of cases included (n). Significant findings after multiple comparison correction are indicated in bold.

**Sample**	**Patient type**	**Phenotype**	**P_T**	**P-value**	**R^2**	**N**
NESDA	Depression and/or anxiety	Loneliness scale	0.1	0.010014	0.38%	1705
GRSD	MDD	Social and family functioning	0.05	0.36	0.07%	1116
STARD	MDD	Social activities and relationships	0.05	0.13	0.2%	1146
CATIE	SCZ	Social withdrawal and social avoidance	0.5	0.053	0.8%	478
STEP-BD	BPD	Interpersonal relations	0.05	**0.00065**	1.6%	701
ROM study	One or more mood, anxiety or somatoform disorders	DAPP	0.001	0.014	1.4%	427
		BSI	0.001	0.334	0.2%	427
		SF36	0.001	0.332	0.2%	427

Supplementary Table 7. Detailed results on annotation of candidate SNPs

[see Excel files for this Table]

Supplementary Table 8. Detailed gene-mapping results

[see Excel files for this Table]
